# Atractylenolide I Inhibits NLRP3 Inflammasome Activation in Colitis-Associated Colorectal Cancer *via* Suppressing Drp1-Mediated Mitochondrial Fission

**DOI:** 10.3389/fphar.2021.674340

**Published:** 2021-07-15

**Authors:** Yao Qin, Yanwei Yu, Chendong Yang, Zhuien Wang, Yi Yang, Chongxu Wang, Qiusheng Zheng, Defang Li, Wenjuan Xu

**Affiliations:** ^1^ School of Integrated Traditional Chinese and Western Medicine, Binzhou Medical University, Yantai, China; ^2^ Yantai Hospital of Traditional Chinese Medicine, Yantai, China

**Keywords:** atractylenolide i, colitis-associated colorectal cancer, NLRP3 inflammasome, mitochondrial fission, DRP1

## Abstract

Inflammatory bowel disease (IBD) is an important high-risk factor that promotes the occurrence and development of colon cancer. Research on the mechanism of regulating NLRP3 can provide potential targets for treating NLRP3 inflammasome–related diseases and changing the inflammatory potential of immune cells. In this study, the effects of atractylenolide I on colitis-associated CRC (caCRC) and inflammasome activation were investigated both *in vivo* and *in vitro*. Furthermore, the role of atractylenolide I on Drp1-mediated mitochondrial fission was analyzed *via* Western blotting and transmission electron microscopy (TEM). Moreover, the Drp1 overexpression lentiviral vector was used to study the role of Drp1 on the signaling mechanisms of atractylenolide I. Atractylenolide I treatment significantly reduced the cell viability of human HCT116 and SW480 cells and induced apoptosis, and effectively inhibited colon tumors in the AOM/DSS mouse model. The reduction of NLRP3 inflammasome activation and excessive fission of mitochondria mediated by Drp1 were associated with the administration of atractylenolide I. Upregulation of Drp1 reversed the inhibitory effect of atractylenolide I on the activation of NLRP3 inflammasomes. Overexpressing the Drp1 expression counteracted the restraint of atractylenolide I on the release of IL-1β of LPS/DSS-stimulated BMDMs. Atractylenolide I inhibited NLRP3 and caspase-1 expression in mice BMDMs, with no influence in the Drp1-overexpressed BMDMs. These results demonstrated that atractylenolide I inhibits NLRP3 inflammasome activation in colitis-associated colorectal cancer *via* suppressing Drp1-mediated mitochondrial fission.

## Introduction

Colorectal cancer (CRC) is one of the most common malignant cancers of the digestive tract, ranking third in the world in incidence of malignant tumors and fourth in the cause of death in 2015 (Siegel et al., 2018). The number of deaths from CRC annually reaches 832,000, and more than half of the cases occur in developed countries (Brody, 2015; Fitzmaurice et al., 2017). Inflammatory bowel disease (IBD) is an important high-risk factor that promotes the occurrence and development of CRC (Peyrin-Biroulet et al., 2011), especially in patients with ulcerative colitis (UC) (Munkholm, 2003). The molecular basis of CRC is the basis of the interplay between host immunity and intestinal flora, intestinal damage, oxidative stress, and chronic inflammation (Beaugerie and Itzkowitz, 2015). Inflammation and cell death initiation by innate immune signaling pathways (including inflammasomes) are the pathogenesis of colitis and colitis-associated CRC (caCRC) (Man, 2018).

NLRP3 (NACHT, LRR, and PYD domain–containing protein 3) inflammasomes are cytoplasmic polyprotein complexes, which are essential for innate immunity. They are composed of a cytoplasmic pattern recognition receptor, NLRP3, the adaptor protein, ASC (apoptosis-associated speck-like protein containing CARD), and caspase-1 (cysteine-requiring aspartate protease-1) (Latz et al., 2013). Particular stimuli from invading pathogens and endogenous “danger signals” (such as extracellular ATP, nigericin, crystals, alum, and amyloid-*β*) can trigger the assembly of inflammasome complexes. After activation, caspase-1 can be cleaved into its activated form (p10 and p20) and promotes the cleavage of pre–IL-1β and pre–IL-18 into mature IL-1β and IL-18 (Latz et al., 2013). Improper activation of NLRP3 inflammasomes is associated with many diseases, such as obesity, UC, Alzheimer’s disease, endotoxin shock, gout, and atherosclerosis (Strowig et al., 2012; Guo et al., 2015). According to reports, NLRP3 protein expression is the limiting step of inflammasome activation (Haneklaus et al., 2013). Therefore, the research on the mechanism of regulating NLRP3 can provide potential targets for treating NLRP3 inflammasome–related diseases and changing the inflammatory potential of immune cells.

Dynamin-related protein 1 (Drp1) is reported to promote membrane fission by contracting and cutting off the mitochondrial membrane by a mechanism that relies on GTP (Tailor et al., 2014). Both the release of cytochrome C and the activation of caspase in the process of apoptosis require its participation (Qian et al., 2012). We detected the expression of Drp1 in tissues and tissue microarrays of CRC patients. The expression of Drp1 in tumor tissues is significantly higher than that in adjacent tissues and normal tissues (Xu et al., 2015). Our results show that Drp1 is closely related to intrinsic apoptosis induction and Bax activation. Regulation of Drp1-mediated mitochondrial fission may be an effective target for cancer treatment (Qian et al., 2013). Mitochondrial elongation caused by the decreased expression of the fission protein Drp1 creates a good cellular environment for ERP-dependent NLRP3 inflammasome activation, which leads to diseases related to inflammatory disorders (Park et al., 2015).

In recent years, naturally occurring chemical substances have attracted attention because they are recognized as cancer-preventive agents with low toxicity and high anti-inflammatory effects. Atractylenolide I (ATR I), the main biologically active ingredient in *Atractylodes macrocephala*, also has a variety of therapeutic activities, including antitumor and anti-inflammatory effects (Long et al., 2020). Atractylenolide I inhibits the occurrence of breast cancer by inhibiting the nuclear factor-κB signaling pathway mediated by toll-like receptor 4 (Long et al., 2020). Atractylenolide I could reduce LPS-induced lung injury in mice (Zhang et al., 2015), inhibit LPS-induced NO release, and reduce the level of pro-inflammatory cytokines in BV-2 cells (More and Choi, 2017). However, the role of atractylenolide I in the activation of NLRP3 inflammasomes and Drp1-mediated mitochondrial fission in CRC remains unknown.

In the present study, the effects of atractylenolide I on caCRC induced by azoxymethane (AOM) and dextran sodium sulfate (DSS) were investigated both *in vitro* and *in vivo*. Furthermore, the role of atractylenolide I on Drp1-mediated mitochondrial fission was analyzed *via* Western blotting and transmission electron microscopy (TEM). Moreover, the Drp1 overexpression lentiviral vector was used to study the role of Drp1 on the signaling mechanisms of atractylenolide I. The present findings suggested that atractylenolide I may be used as a novel probiotic product for the treatment of CRC.

## Materials and Methods

### Cell Culture, Animals, and Reagents

Human colon cell lines (HCT116 and SW480) were obtained from the American Type Culture Collection (ATCC, Rockville, MD). All cell lines were incubated in RPMI-1640 (Invitrogen) and DMEM (Invitrogen), supplemented with 10% (v/v) fetal bovine serum (Invitrogen) and 1% (v/v) penicillin–streptomycin (Invitrogen) at 37°C in a humidified atmosphere of 5% CO_2_. C57BL/6J male mice (5–6 weeks old, 18–22 g) were purchased from the the Animal Supplier Center of Binzhou Medical University. All procedures involving laboratory animals were in accordance with the guidelines of the Instituted Animal Care and Use Committee of Binzhou Medical University. All protocols were submitted and validated by the Animal Care Ethics Committee of Binzhou Medical University. Bone marrow–derived macrophage (BMDM) cells were isolated according to the following procedures. Bone marrow cells were isolated from C57/BL6 mice and cultured with DMEM supplemented with 10% fetal bovine serum and 20 ng/ml GM-CSF (Peprotech, Rock Hill, NJ). The culture fluid was exchanged to a fresh culture medium every 3 days. Under these conditions, adherent macrophages were obtained within 7–8 days. Cells were harvested and seeded on 24-well plates. After culture for 6 h without GM-CSF, the cells were used for the experiments as bone marrow–derived macrophages.

Atractylenolide I was obtained from Chengdu Herbpurify CO. Ltd. (Chengdu, China), dissolved in dimethylsulfoxide (DMSO; Sigma-Aldrich, St. Louis, MO, United States), at 100 mM stock and diluted immediately before each experiment.

### Measurement of Cell Viability

Cell viability assays were performed using the Cell Counting Kit-8 (CCK-8; Dojindo Molecular Technologies, Japan). The cells were inoculated in a 96-well plate with culture medium and incubated at 37°C for 24 h. After acclimatization, the cells were treated with atractylenolide I for 24 and 48 h. Then the medium was replaced with fresh medium containing 10 ml of the CCK-8 solution. After incubating for 2 h at 37°C, the optical density (OD) at 490 nm was measured.

### ROS Analysis, Apoptosis Assay, and MMP Assay by Flow Cytometry

Cells were treated with atractylenolide I for 24 h, and then the cells were processed by ROS analysis. In brief, 5 × 10^4^ cells were suspended in 10 μmol/L of DCFH-DA solution and incubated for 30 min in the dark according to the manufacturer’s instructions. The ROS level was analyzed by FACS flow cytometry.

Cells were stained with a Annexin V-FITC Apoptosis Detection Kit (BD Biosciences, San Jose, CA, United States). According to the manufacturer’s instructions, the cells were incubated with 5 ml of Annexin V and 5 ml of propidium iodide (PI) for 15 min at room temperature, and then the stained cells were analyzed on a FACS flow cytometer.

Cells were treated with atractylenolide I for 24 h, and then the cells were performed by a mitochondrial membrane potential (MMP) assay. In brief, cells were suspended in 2 μmol/L of JC-1 solution and incubated for 30 min in the dark according to the manufacturer’s instructions. The MMP level was analyzed by FACS flow cytometry.

### Protein Expression Analysis

Protein expression was analyzed by Western blot. The total protein (50 μg) was separated by sodium dodecyl sulfate-polyacrylamide gel electrophoresis. After the protein is transferred to the polyvinylidene fluoride microporous membrane (Bio-Rad), the membrane is blocked with 5% skimmed milk powder and the primary antibody [anti-Bax, anti-PCNA, anti–*β*-catenin, anti-Drp1 (1:1,000 dilution, American Abcam), anti-NLRP3 (1:500 dilution, American Abcam), anti-ASC, or anti–caspase-1]; then anti-mouse (anti-rabbit (IgG)) is linked with fluorescein (1: 1,000), and then incubated with an antibody conjugated to anti-fluorescein alkaline phosphatase (1:5,000). The immune complexes were detected with enhanced chemiluminescence reagents. For quantification, the signal optical density was normalized to *β-*actin by Quantity One image analysis software.

### Measurement of *in Vivo* Activity

The animals were kept under controlled conditions in a regular animal community (22°C, 12 h/12 h dark/light cycle). At the beginning of the experiment, the mice were between 5 and 8 weeks old and weighed 15–22 g. 46 mice were used in the study, 10 mice were in the normal control group (control group), 20 mice were in the AOM/DSS (model) group, and 16 mice were in the AOM/DSS and atractylenolide I group. At the end of the study, there were 10 surviving mice in the control group, 12 surviving mice in the model group, and 12 surviving mice in the atractylenolide I group. DSS is shown in Figure 3A. On day 1, C57BL/6J mice were injected with AOM (10 mg·kg-1, i.p.). After 5 days, 4% DSS (International Laboratory, Chicago, Illinois, United States) was added to drinking water for 7 days, and then tap water was added for another 14 days for recovery. This cycle was repeated twice. Atractylenolide I (25 mg/kg, 50 mg/kg) or the equivalent concentration of normal saline was added twice a day with a gavage tube. At the 10th week, the mice were killed by cervical dislocation, and the colon was removed (from the ileocecal junction to the edge of the anus). Record the number, size, and location of preneoplastic and neoplastic lesions (dysplasia and cancer) in the colon according to the gross examination. Measure the size of dysplasia by a visual micrometer. After a rough inspection, the colon was cut into small pieces at approximately 1 cm intervals and stored at −80°C for further immunohistochemistry and protein analysis.

### Histopathological Assessment

For histopathological examination, the formalin-fixed and paraffin-embedded colon tissue was cut into serial sections (5 μm) and stained with hematoxylin and eosin (H& E). Histological changes were confirmed, such as dysplasia and cancer. The assessment of dysplasia and adenocarcinoma was based on the criteria proposed in the mice Models of Intestinal Cancers consensus report. The height of the intestinal villi is the distance from the base of the villi (the junction between the intestinal glands and the villi) to the top of the villi. Intestinal villi were measured using the Image-pro plus 6.0 image analysis software.

### Immunohistochemistry

The paraffin-embedded colon sections were deparaffinized, rehydrated, and pretreated with hydrogen peroxide in a PBS buffer. Heat-induced antigen retrieval was performed. The sections were incubated with anti-Drp1 (1:1,000 dilution, Abcam, United States) and anti-NLRP3 (1:100 dilution, Abcam, United States). The sections were incubated with HRP-conjugated secondary antibody, tyramide amplification was performed first, streptavidin-HRP was added, and then the DAB kit was used to show a positive signal. The sections were inspected at 400× magnification and analyzed using NIS-Elements. The following formula was used to calculate the positive content: positive content (PC) = average optical density x positive area.

### Measurement of IL-1β in the Serum of Mice by ELISA

The mice in the control group and the treatment group were sacrificed, and the mice serum was collected. The concentration of IL-1β in serum was determined by ELISA (Ebiosciences-Easy-Set-Go) according to the manufacturer’s protocol.

### PLVX-IRES-ZsGreen1-Drp1 Expression Vector Construct

The pLVX-IRES-ZsGreen1 lentivirus vector was purchased from Genomeditech (Shanghai, China). To clone human Drp1 cDNA into the pLVX-IRES-ZsGreen1 vector, XhoI and NotI restriction sites were inserted into the PCR products. After confirming the Drp1 sequences by sequencing, the plasmid was cotransfected into 293T cells with the lentivirus packaging plasmids pMD2.G and psPAX2 to produce lentivirus particles. The Drp1 lentivirus particles were then used to infect mice BMDMs, and the control and AOM/DSS mice models. Four weeks after the mice were modeled, Drp1-overexpressed lentivirus was injected into the tail vein. The lentivirus titer was 1 × 10^8^, and the injection dose was 100 μl, it was injected only once. The mice model and BMDMs stably overexpressing Drp1 or empty vector were established by first transducing the cells with Drp1 lentivirus particles or empty vector lentivirus particles.

### mROS Production

To determine the production of mROS, cells were stained with MitoSOX (Invitrogen) according to the manufacturer’s protocol. Then the fluorescence of the cells was monitored by a flow cytometer (FACSVerse, BD).

### Statistical Analysis

Each experiment was repeated at least three times. Data were expressed as mean ± SD. All data were analyzed using SPSS statistical software package (version 23.0, SPSS Inc., Chicago, Illinois, United States). The data were compared between the two groups with two independent sample tests. The average of data from more than three groups was compared with one-way analysis of variance (ANOVA), and multiple comparisons were made. A value of *p* < 0.05 was considered statistically significant.

## Results

### Atractylenolide I Significantly Inhibits Cell Viability *via* Apoptosis to a Certain Extent in CRC Cells

**FIGURE 1:**
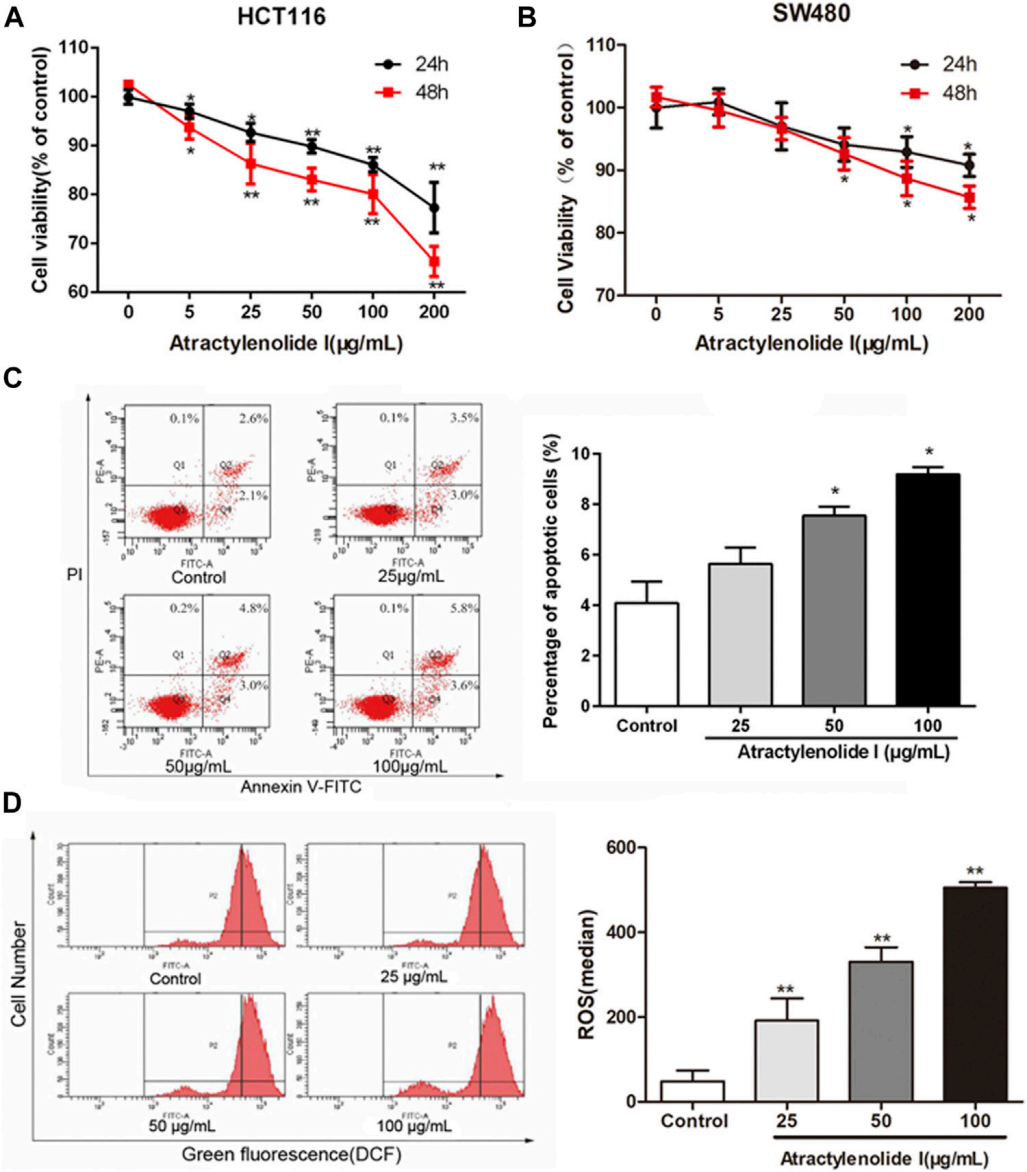
Atractylenolide I significantly inhibit cell viability *via* apoptosis to a certain extent in CRC cells. **(A)** Cell viability treated with different concentrations of atractylenolide I was measured by the CCK-8 assay at 24 and 48 h and plotted as a dose–response curve relative to no-drug controls (*n* = 6 per group). **(B)** SW480 cells treated with the atractylenolide I (n = 6 per group). **(C)** Measurement of PE-Annexin V staining of phosphatidylserine exposed on the cell surface by flow cytometry (n = 3 per group). **(D)** The ROS levels in HCT-116 cells treated with atractylenolide I (25, 50, and 100 μg/ml) by loading DCFH-DA fluorescent probes (n = 3 per group). ^*^ *p* < 0.05; **p < 0.01, vs. control (mean ± S.D).

To address the role of atractylenolide I on the cell viability, HCT116 and SW480 (CRC cell lines) were treated with different concentrations of atractylenolide I (0–200 μg/ml). As shown in Figure 1, atractylenolide I significantly reduced the viability of HCT116 cells in a dose- and time-dependent manner with an IC50 value of 630 (atractylenolide I) μg/mLfor 24 h (Figure 1A). The results also indicated that 200 μg/ml of atractylenolide I was weakly cytotoxic (10–15% inhibition rate) for SW480 cell (Figure 1B). Thus, HCT116 cells were used in subsequent experiments to study the mechanism of atractylenolide I. Annexin V/propidium iodide (PI) double staining (Figure 1C) showed that most of the cell deaths in HCT116 induced by atractylenolide I could be attributed to apoptosis. But in terms of inhibiting cell proliferation, the ability of atractylenolide I was a relatively low degree.

### Atractylenolide I Increases ROS Content and Induces the Changes in Mitochondrial Function of CRC Cells

We detected the changes in ROS levels in HCT-116 cells treated with atractylenolide I (25, 50, and 100 μg/ml) by loading DCFH-DA fluorescent probes. The analysis of the results of the fluorescence intensity of DCF detected by flow cytometry showed that compared with the control group, the intracellular ROS content in the atractylenolide I groups was significantly increased (Figure 1D).

**FIGURE 2:**
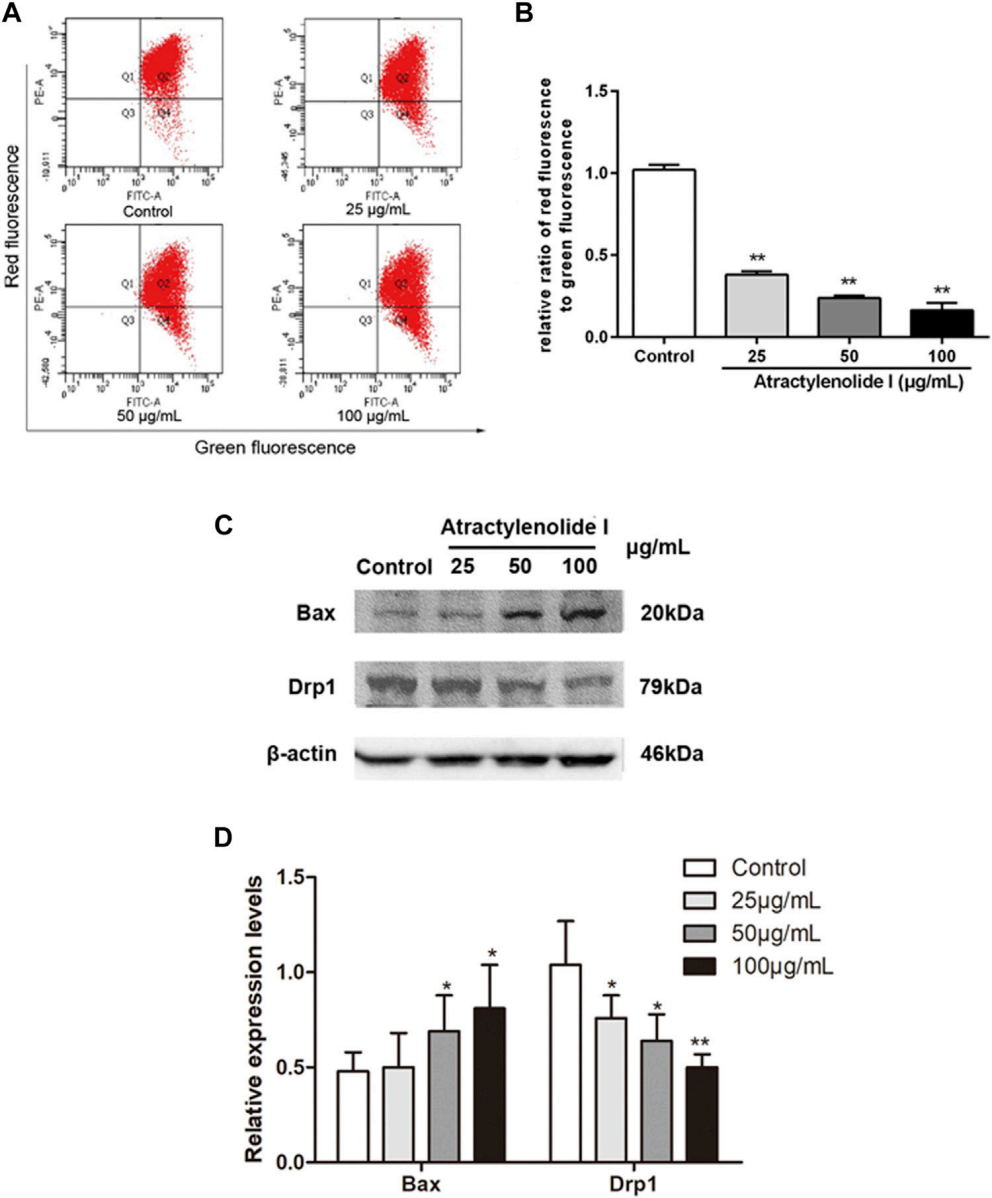
Atractylenolide I increases ROS content and induces the changes in mitochondrial function of CRC cells. **(A,B)** HCT116 cells were treated with atractylenolide I for 24 h and then stained with the fluorescent dye JC-1. The mitochondrial membrane potential (MMP) was measured with a flow cytometer, which showed the distribution of red fluorescence (JC-1 aggregate) and green fluorescence (JC-1 monomer) in cells. The mitochondrial membrane potential was quantitatively analyzed as the means ± S.D. **(C)** HCT116 cells were treated atractylenolide I for 24 h. **(D)** Extract total cell lysate for Western blot analysis of Bax and Drp1 (n = 3 per group). The densitometric analysis bar diagram of the results. ^*^ *p* < 0.05; **p < 0.01, vs. control (mean ± S.D).

We used the JC-1 method to detect the integrity of the mitochondrial membrane, where red and green fluorescence represent the normal mitochondria and the damaged mitochondria, respectively. Figures 2A,B showed a dose-dependent decrease in the ratio of red/green fluorescence in the cells treated with atractylenolide I, indicating that atractylenolide I could induce mitochondrial dysfunction and apoptosis. Atractylenolide I increased the expression of Bax in HCT116 cells. Reduction of Drp1 was observed by atractylenolide I in HCT116 cells (Figures 2C,D). Combined with the previous research in CRC cells, the dissipation of ΔΨm and the activation of Bax and downregulation of Drp1 indicated that the cell death induced by atractylenolide I could be regarded as intrinsic apoptosis, and atractylenolide I might inhibit the Drp1-mediated mitochondrial fission.

### Atractylenolide I Reduced the Incidence and Multiplicity of Colonic Neoplasms of Mice Treated With AOM/DSS

**FIGURE 3:**
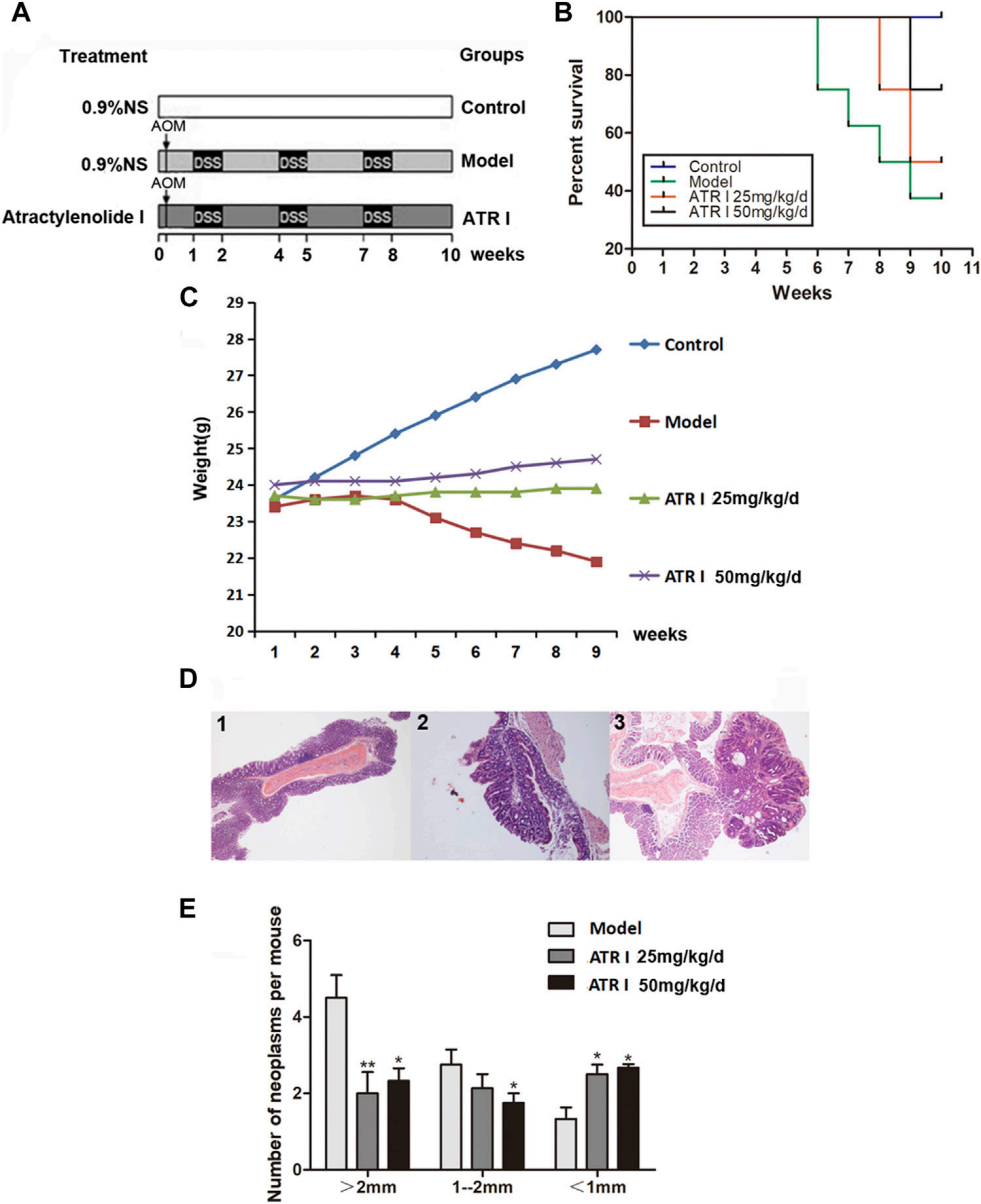
Atractylenolide I reduced the incidence and multiplicity of colonic neoplasmsof mice treated with AOM/DSS. **(A)** Experimental protocol for the mice model of colon cancer associated with colitis. **(B)** Effect of atractylenolide I on the survival ratio of mice. **(C)** The effect of atractylenolide I on the weight of mice (Control: 25.82 ± 1.41; Model: 22.95 ± 0.68, *p*-value 0.0001). **(D)** Histological study based on hematoxylin and eosin (H&E) staining. 40× for all, scale bar = 100 μm. 1: control mice; 2–3: model mice. **(E)** Effect of atractylenolide I on the colonic neoplasm size. ^*^ *p* < 0.05; **p < 0.01, vs. model (mean ± S.D).

AOM/DSS was used to construct the mice model of caCRC, and the effect of atractylenolide I on caCRC was observed (Figure 3A). The survival rate was observed in mice exposed to AOM/DSS (Figure 3B). Atractylenolide I significantly delayed mass death in the AOM/DSS mice model (*p*-value 0.0338). A dose gradient of atractylenolide I (25 mg/kg, 50 mg/kg) was prescreened. The body weight fluctuation was substantially alleviated in mice receiving atractylenolide I treatment (25 mg/kg/d: 23.76 ± 0.11, *p*-value 0.0028; 50 mg/kg/d: 24.29 ± 0.25, *p*-value 0.0000) (Figure 3C). As shown by the survival rate and body weight of the mice, atractylenolide I was well tolerated in mice, and no obvious systemic toxicity was observed during the entire course of drug treatment. Most neoplasms were disseminated in the middle and distal colon, and were confirmed histologically (Figure 3D). Atractylenolide I treatment significantly reduced the number of large tumors (>2 mm). The number of neoplasms larger than 2 mm in atractylenolide I treatment group was 30.77% less than that in the untreated mice (Figure 3E).

### Atractylenolide I Inhibited NLRP3 Inflammasome Formation and Activation Induced IL-1β Secretion

**FIGURE 4:**
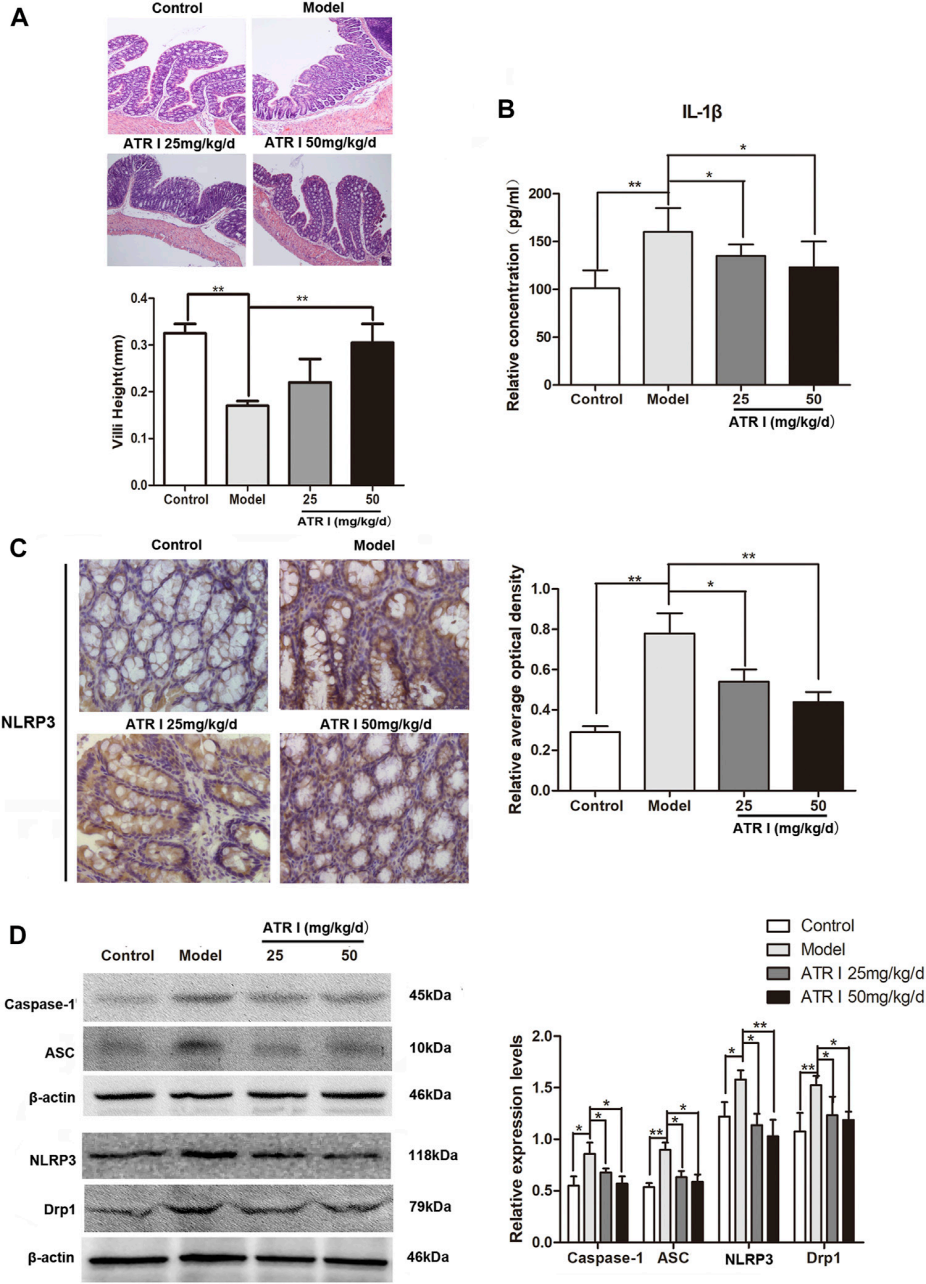
Atractylenolide I inhibited NLRP3 inflammasome formation and activation induced IL-1β secretion. **(A)** Histological study based on hematoxylin and eosin (H&E) staining, scale bar = 200 μm. Villi height was measured. **(B)** The concentrations of IL-1β in the serum of mice were measured by ELISA. **(C)** IHC of NLRP3 in colonic tissue section. Positive staining is brown. **(D)** The expression levels of NLRP3, caspase-1, ASC, and Drp1 in the intestinal tract were detected by the Western blot analysis (n = 3 per group). The densitometric analysis bar diagram of the results: ^*^ *p* < 0.05; **p < 0.01, vs. model (mean ± S.D).

H&E staining showed congestion of intestinal mucosa, massive loss of surface epithelium, and unclear colonic villi in the AOM/DSS mice model group. Colonic villi were abundant in the atractylenolide I group, and the height was significantly increased (Figure 4A). The NLRP3 inflammasomes have been shown to play a key role in AOM/DSS-induced caCRC model (Dai et al., 2020). To determine whether atractylenolide I inhibits NLRP3 inflammasomes *in vivo*, we evaluated the expression of NLRP3 in the AOM/DSS mice model by immunohistochemistry (IHC) and Western blotting. Atractylenolide I similarly reduced the increased secretion of IL-1β in serum (Figure 4B). In addition, atractylenolide I significantly reduced the accumulation of NLRP3 in the intestine (Figure 4C). Atractylenolide I also significantly reduced the expression of intestinal NLRP3, caspase-1, and ASC induced by AOM/DSS (Figure 4D). Overall, the results showed that atractylenolide I inhibited the activated NLRP3 inflammasomes in the AOM/DSS mice model.

### Atractylenolide I Inhibits Drp1-Mediated Mitochondrial Fission in the AOM/DSS Mice Model

**FIGURE 5:**
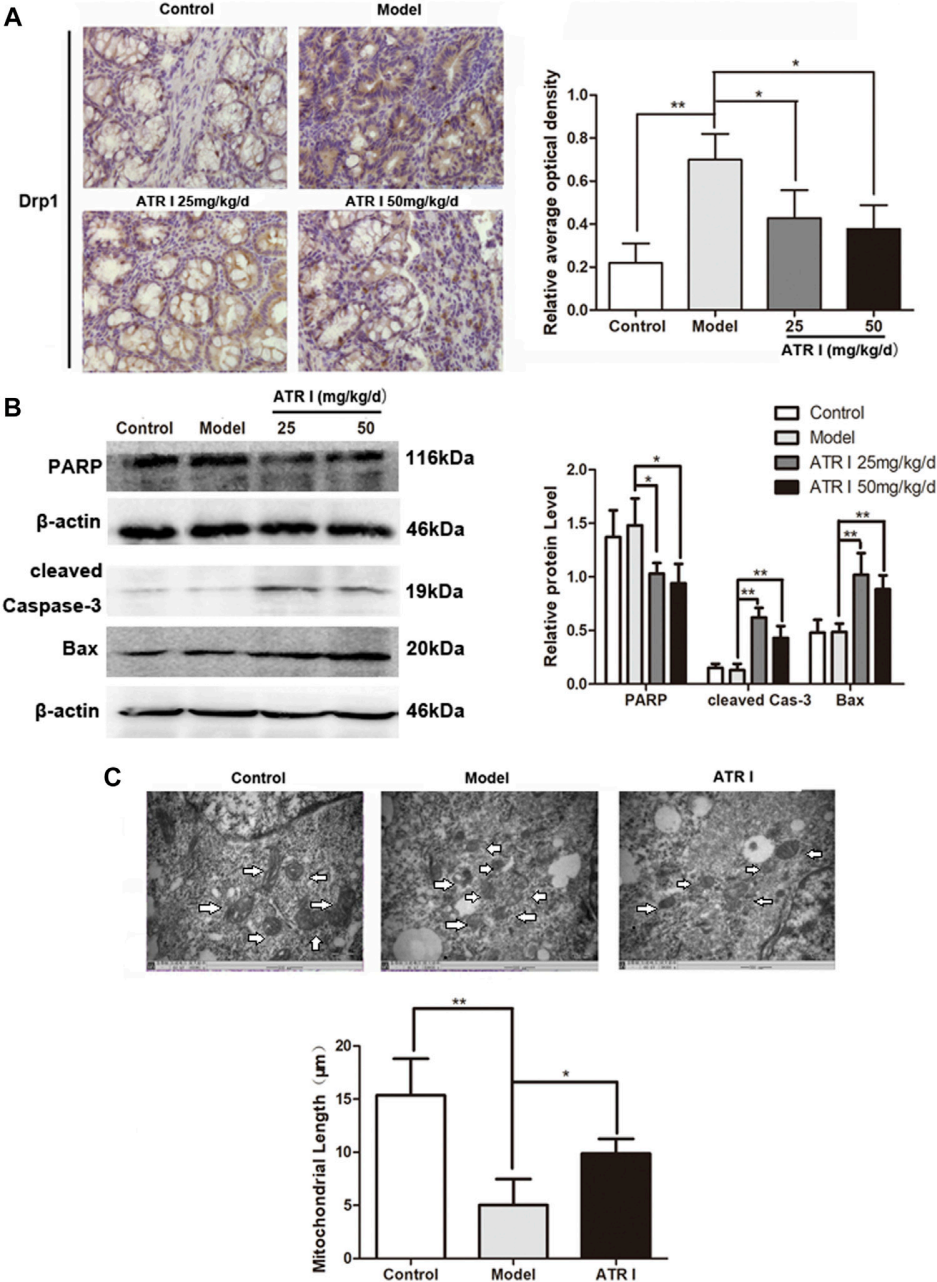
Atractylenolide I inhibits Drp1-mediated mitochondrial fission in the AOM/DSS mice model. **(A)** IHC of Drp1 in colonic tissue section. Positive staining is brown. **(B)** Extract intestinal tissue lysate for Western blot analysis of PARP, cleaved caspase-3, and Bax (n = 3 per group). The densitometric analysis bar diagram of the results. **(C)** TEM was applied to observe the morphology of the mitochondria. Mitochondrial length was representative of three independent experiments. ^*^ *p* < 0.05; **p < 0.01, vs. model (mean ± S.D).

The mitochondrial dysfunction was further confirmed by the decreased mitochondrial membrane potential and the expression level of Drp1 proteins of atractylenolide I–treated HCT116 cells. Atractylenolide I significantly reduced the expression of mitochondrial fission marker, Drp1, in the AOM/DSS mice model by Western blotting (Figure 4D) and immunohistochemistry (Figure 5A). The immunoblotting assessment (Figure 5B) showed that the cleavage of poly (ADP) ribose polymerase (PARP), caspase-3, and Bax was also increased in the AOM/DSS mice model, which were treated with atractylenolide I. In addition, the morphology of the mitochondria detected by TEM showed a weak mitochondrial fission ability in a atractylenolide I-treated AOM/DSS mice model (Figure 5C), suggesting that atractylenolide I inhibited mitochondrial fission to induce apoptosis in the AOM/DSS mice model.

### Upregulation of Drp1 Reverses the Effect of Atractylenolide I on NLRP3 Inflammasome Activation

**FIGURE 6:**
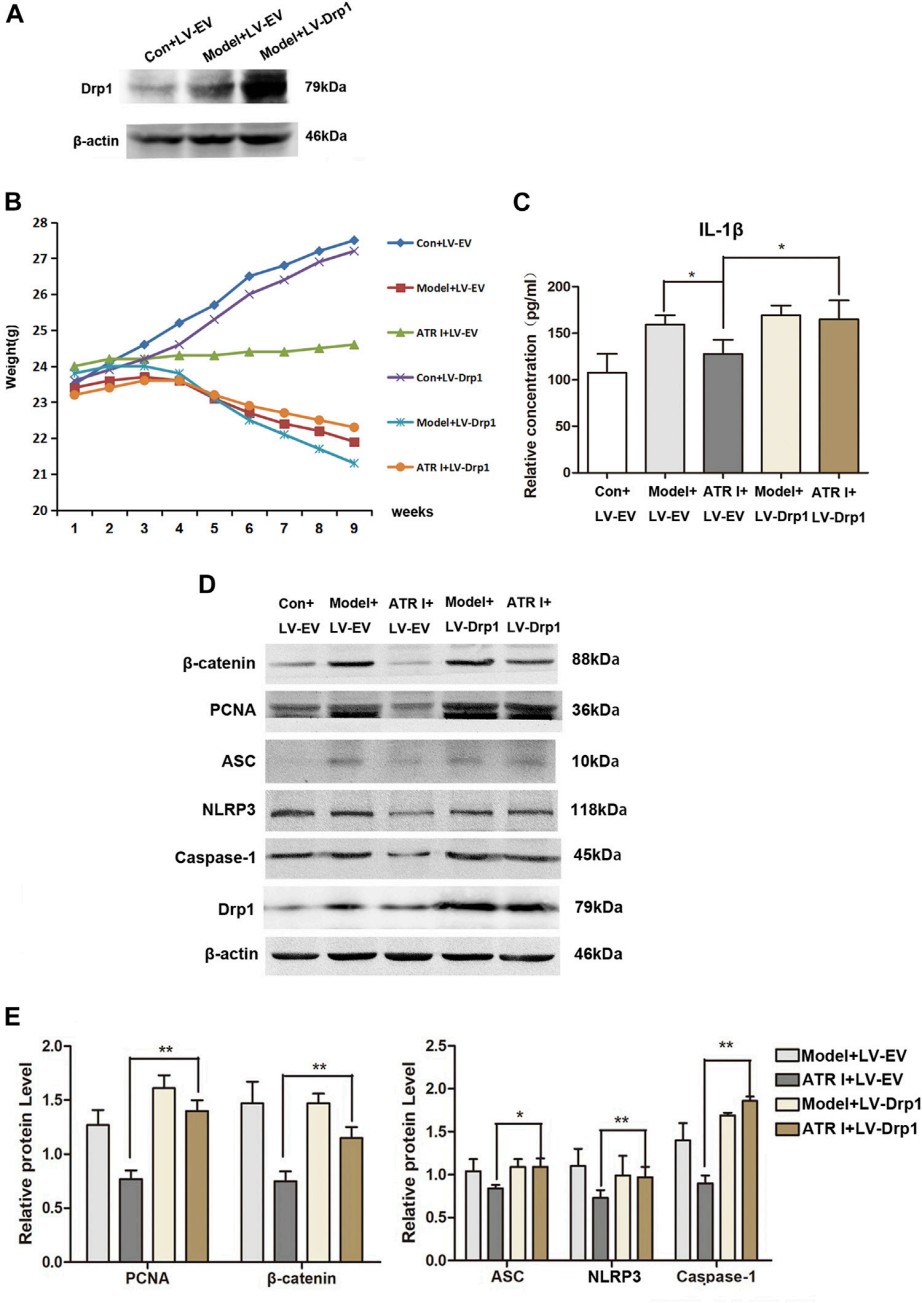
Upregulation of Drp1 reverses the effect of atractylenolide I on NLRP3 inflammasome activation. **(A)** Drp1 lentivirus (LV-Drp1) expression vector transduction increased the expression of Drp1 compared to that of empty vector (EV) transduction. **(B)** Effect of atractylenolide I on body weight of mice transfected with the Drp1 lentivirus vector or empty vector (Model + LV-Drp1: 22.92 ± 1.05 vs ATR I + LV-Drp1: 23.04 ± 0.47, *p*-value 0.7584). **(C)** The concentrations of IL-1β in the serum of mice were measured by ELISA. **(D,E)** Expression levels of NLRP3, caspase-1, ASC, *β*-catenin, and PCNA in the intestinal tract were detected by the Western blot analysis (n = 3 per group). The densitometric analysis bar diagram of the results: ^*^ *p* < 0.05; **p < 0.01, vs. model (mean ± S.D).

In order to determine whether Drp1 was involved in the activation of the NLRP3 inflammasomes, the overexpression of Drp1 in control and AOM/DSS mice model transduced with the Drp1 lentivirus vector were established (Figure 6A). We found that the body weight fluctuation was not substantially alleviated in mice receiving atractylenolide I treatment, which were transduced with the Drp1 lentivirus vector (ATR I + LV-EV: 24.32 ± 0.18 vs ATR I + LV-Drp1: 23.04 ± 0.47, *p*-value 0.0000) (Figure 6B). In addition, overexpressing the Drp1 expression counteracted the restraint of atractylenolide I on the release of IL-1β (Figure 6C). Furthermore, transducing with the Drp1 lentivirus vector significantly upregulated β-catenin and PCNA expression in the atractylenolide I–treated AOM/DSS mice model (Figures 6D,E). Upregulation of Drp1 reversed the inhibitory effect of atractylenolide I on the activation of NLRP3 inflammasomes (Figures 6D,E). Collectively, these results indicated that Drp1 was related to the regulation of atractylenolide I on the activation of NLRP3 inflammasomes in the AOM/DSS mice model.

### Atractylenolide I Inhibited NLRP3 Inflammasome Activation by a Drp1-dependent Manner in LPS/DSS-Stimulated Mice Bone Marrow–Derived Macrophages (BMDMs)

**FIGURE 7:**
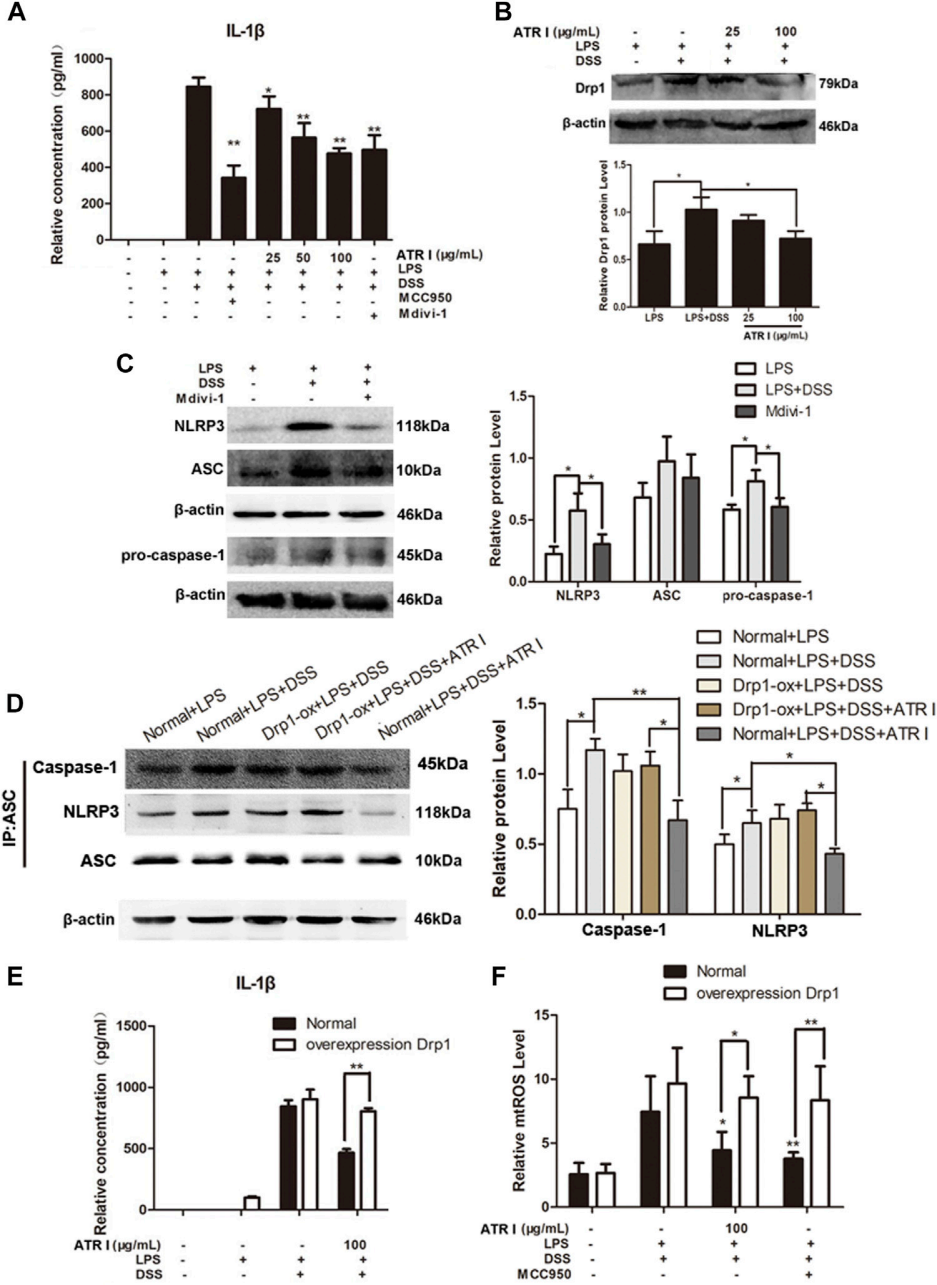
Atractylenolide I inhibited NLRP3 inflammasome activation by a Drp1-dependent manner in LPS/DSS-stimulated mice BMDMs. To generate macrophages with the overexpression of Drp1, Drp1 lentivirus expression vector transduction and empty vector transduction were used to infect BMDMs. Mice BMDMs were pretreated with PBS or atractylenolide I for 2 h and then stimulated with 5% DSS and LPS (100 ng/ml) for indicated time periods. **(A)** The expression of IL-1β in the culture supernatants was measured by ELISA. **(B)** The expression levels of Drp1 were detected by the Western blot analysis (n = 3 per group). **(C)** Expression levels of NLRP3, ASC, and pro–caspase-1in BMDMs treated with Mdivi-1 were detected by the Western blot analysis. (n = 3 per group). The densitometric analysis bar diagram of the results. **(D)** Cell lysates were immunoprecipitated with anti-ASC. The NLRP3 and caspase-1 expression were detected by the Western blot analysis. **(E)** Overexpressing Drp1 expression counteracted the restraint of atractylenolide I on the release of IL-1β of LPS/DSS-stimulated BMDMs. **(F)** Cells were stained with MitoSOX and then analyzed by flow cytometry (n = 3 per group). The densitometric analysis bar diagram of the results: ^*^ *p* < 0.05, **p < 0.01 vs. Normal + LPS + DSS + ATR I (mean ± S.D).

In order to gain insights into the mechanism of the anti-inflammatory effect of atractylenolide I, we studied the effect of atractylenolide I on the activation of NLRP3 inflammasomes *in vitro*. As shown in Figure 7A, atractylenolide I showed a significant dose-dependent inhibitory effect on the activation of IL-1β in BMDMs stimulated by LPS/DSS. LPS/DSS-stimulated secretion of IL-1β was inhibited by Drp1 inhibitor Mdivi-1, as well as MCC950, a potent and selective small molecule inhibitor, and can normally and atypically inhibit NLRP3 activation. Also, atractylenolide I significantly reduced the expression of Drp1 in BMDMs stimulated by LPS/DSS (Figure 7B). Mdivi-1 treatment reduced the expression of NLRP3, ASC, and pro–caspase-1 in BMDMs stimulated by LPS/DSS (Figure 7C). To generate macrophages with the overexpression of Drp1, the Drp1 lentivirus (LV-Drp1) expression vector transduction and empty vector (EV) transduction were used to infect BMDMs. At the protein immunoprecipitated with anti-ASC, atractylenolide I greatly inhibited the expression of NLRP3 and caspase-1, but it had no effect on the expression of NLRP3 and caspase-1 in atractylenolide I–treated Drp1 overexpression BMDMs (Figure 7D). Overexpressing Drp1 expression counteracted the restraint of atractylenolide I on the release of IL-1β of LPS/DSS-stimulated BMDMs (Figure 7E). Taken together, these data indicated that atractylenolide I could inhibit the NLRP3 expression and subsequent IL-1β secretion induced by NLRP3 inflammasome activation in a Drp1-dependent manner.

We next detected the effect of atractylenolide I and MCC950 on the mitochondrial ROS level. Atractylenolide I and MCC950 decreased the level of mtROS induced by the addition of LPS/DSS *in vitro*. Of more interest, after LPS/DSS stimulation activated NLRP3, the level of mtROS in Drp1 overexpression BMDM was significantly higher than that in control BMDM (Figure 7F). These findings increased the possibility that Drp1-mediated mitochondrial fission might play an effective role in NLRP3 inflammasome activation.

## Discussion

Epidemiological studies have shown that IBD is an important high-risk factor for colon cancer (Noti et al., 2010; Danese et al., 2011). Due to the long course of inflammatory bowel disease and easy recurrence or no effective treatment, inflammation repeatedly stimulates the lesions for a long time, leading to the development of colorectal cancer (Nielsen et al., 2013). Anti-inflammatory drugs have become a drug of concern to scientific researchers, and they have begun to explore whether anti-inflammatory drugs can effectively prevent the occurrence of colorectal cancer.

Here, we introduce an effective and safe method to alleviate acute colitis and reduce caCRC through atractylenolide I treatment. Atractylenolide I is a highly effective active ingredient in *Atractylodes macrocephala*. It is also combined with *Atractylodes macrocephala* for the adjuvant treatment of cancer in clinical applications. Researchers have conducted pharmacological studies on it (Liu et al., 2016; Zhu et al., 2018). Studies have shown that atractylenolide I has obvious inhibitory effects on a variety of tumor cells. For example, atractylenolide I can inhibit the proliferation of bladder cancer cell line (Yu et al., 2016). Atractylenolide I inhibits the growth of lung cancer cells by inhibiting PDK1 and LncRNA HOTAIR-mediated EZH2 gene expression (Xiao et al., 2018). Atractylenolide I can also promote ovarian cancer cell cycle arrest and apoptosis by inhibiting the PI3K/Akt/mTOR pathway (Long et al., 2017). Researchers found that atractylenolide I can restore the expression of HO-1 and inhibit the proliferation, migration, and inflammatory response of VSMC induced by Ox-LDL *in vitro*. (Li et al., 2017). This study demonstrated that atractylenolide I reduced cell viability and induced cell apoptosis. But in terms of cytotoxicity and apoptotic induction, the ability of atractylenolide I was to a relatively low degree. However, atractylenolide I exhibited more potent antitumor effects in increasing intracellular ROS and reducing mitochondrial membrane potential. Combined with the previous research in CRC cells, the dissipation of ΔΨm, the activation of Bax, and downregulation of Drp1 suggest that the cell death induced by atractylenolide I could be regarded as intrinsic apoptosis, and atractylenolide I might inhibit the Drp1-mediated mitochondrial fission. Mitochondrial dysfunction has a major impact on cell apoptosis, metabolism, and cancer development. Emerging evidence shows that the dynamic balance between mitochondrial fission and fusion is essential for maintaining the shape, distribution, and function of the mitochondria (Galluzzi et al., 2012; Zhao et al., 2012). Among the key molecules, the mitochondrial fission marker motility-related protein 1 (Drp1) is the most important (Trotta and Chipuk, 2017). It has been reported in the literature that the overexpression of Drp1 could induce a liver cancer mitochondrial division and promote cell survival (Senft and Ronai, 2016; Zhou et al., 2017; Hagenbuchner et al., 2019). The inhibition of Drp1 activity could significantly inhibit cancer cell growth and metastasis (Rehman et al., 2012). However, the basic mechanism of Drp1 regulation remains to be elucidated, which is essential for targeting atractylenolide I to mitochondrial fission.

This study demonstrated that atractylenolide I significantly suppressed the formation of colonic neoplasms and delayed mass death in the AOM/DSS mice model. The appropriate activation of NLRP3 inflammasomes is essential for immune defense, but improper activation could cause inflammatory diseases. Previous studies have shown that the NLRP3 inflammasome exacerbates the severity of acute colitis induced by DSS (Stehlik et al., 2003; Sun et al., 2015) and inherited spontaneous colitis (Zhang et al., 2014; Zmora et al., 2017). Based on this, our study here characterized that atractylenolide I is a safe and effective drug for the treatment of AOM/DSS-induced caCRC, and its mechanism is mainly to inhibit the activation of Drp1 and NLRP3 inflammasomes. The expression of NLRP3, caspase-1, and ASC, and the release of IL-1β were all suppressed by atractylenolide I. Furthermore, transfection with the Drp1 lentivirus vector significantly upregulated β-catenin and PCNA expression in the atractylenolide I–treated AOM/DSS mice model. Upregulation of Drp1 reversed the inhibitory effect of atractylenolide I on the activation of NLRP3 inflammasomes. Collectively, these results indicated that Drp1 was involved in the regulation of the activation of NLRP3 inflammasomes by atractylenolide I in the AOM/DSS mice model. Increased production of pro-inflammatory cytokines is a hallmark of DSS-induced colitis (Egger et al., 2000). Among these cytokines, IL-1β plays an important role in intestinal inflammation (Coccia et al., 2012). In our study, compared with the effect of the NLRP3 inhibitor MCC950, atractylenolide I successfully reduced the high expression of IL-1β in BMDMs induced by LPS and DSS in a dose-dependent manner. The production of IL-1β requires the activation of caspase-1, which depends on the activated inflammasomes to convert pro–IL-1β into its mature active form. Therefore, inflammasomes are thought to mediate the host’s defense against microbial pathogens and maintain intestinal homeostasis, thereby contributing to inflammatory diseases and colon cancer (Chen and Nunez, 2011). Although there are many types of inflammasomes, NLRP3 inflammasomes are the most extensively studied and most complex caspase-1 inducers in intestinal inflammation and colon tumors (Villani et al., 2009). At the same time, we found that Mdivi-1, an inhibitor of mitochondrial fission, could also reduce the level of IL-1β in the cell supernatant, indicating that mitochondrial fission may be involved in DSS-induced inflammasome activation. In BMDMs extracted from Drp1 overexpressing mice infected with lentivirus, atractylenolide I did not significantly decrease the IL-1β content, further indicating that atractylenolide I reduces the activation of NLRP3 inflammasomes by inhibiting the excessive fission of mitochondria mediated by Drp1. After overexpression of Drp1, atractylenolide I treatment greatly inhibited the expression of NLRP3 and caspase-1 at the protein level of mouse BMDMs, but it had no effect on the expression of NLRP3 and caspase-1 in the overexpression of Drp1 treated with atractylenolide I in BMDM. In summary, these data indicated that atractylenolide I could inhibit the NLRP3 expression and subsequent IL-1β secretion induced by NLRP3 inflammasome activation in a Drp1-dependent manner.

In conclusion, these data demonstrated that atractylenolide I treatment significantly reduced the cell viability of human HCT116 and SW480 cells and induced their apoptosis, and effectively inhibited colonic neoplasms in the AOM/DSS mice model. The decrease of NLRP3 inflammasome activation and excessive fission of mitochondria mediated by Drp1 were associated with atractylenolide I administration. Atractylenolide I inhibits NLRP3 inflammasome activation in colitis-associated colorectal cancer *via* suppressing Drp1-mediated mitochondrial fission. We used MitoSOX to label mitochondrial ROS (mtROS). Atractylenolide I could reverse the mitochondrial damage caused by the activation of NLRP3 inflammasomes. ROS may be a key element for Drp1-mediated mitochondrial fission and the activation of NLRP3 inflammasomes. Follow-up experiments need to continue to study its mechanism of action.

## Data Availability

The raw data supporting the conclusions of this article will be made available by the authors, without undue reservation.

## Supplementary Material

Supplementary Figure 1-4 Histological study based on H&E staining (The original image of Figure 4A). 1:Control, 2:Model, 3:ATR I 25 mg/kg/d, 4:ATR I 50 mg/kg/d.

Supplementary Figure 5-8 IHC of NLRP3 in colonic tissue section. Positive staining is brown (The original image of Figure 4C). 5:Control, 6:Model, 7:ATR I 25 mg/kg/d, 8:ATR I 50 mg/kg/d.

Supplementary Figure 9-12 IHC of Drp1 in colonic tissue section. Positive staining is brown (The original image of Figure 5A). 9:Control, 10:Model, 11:ATR I 25 mg/kg/d, 12:ATR I 50 mg/kg/d.

Supplementary Figure 13-15 TEM was applied to observe the morphology of the mitochondria (The original image of Figure 5C). 13:Control, 14:Model, 15:ATR I.
