# Supplementary figures and images for: Atractylenolide I Inhibits NLRP3 Inflammasome Activation in Colitis-Associated Colorectal Cancer via Suppressing Drp1-Mediated Mitochondrial Fission
Source: Front Pharmacol. 2021 Jul 15;12:674340. doi: 10.3389/fphar.2021.674340 (PMC8320763; doi:10.3389/fphar.2021.674340)

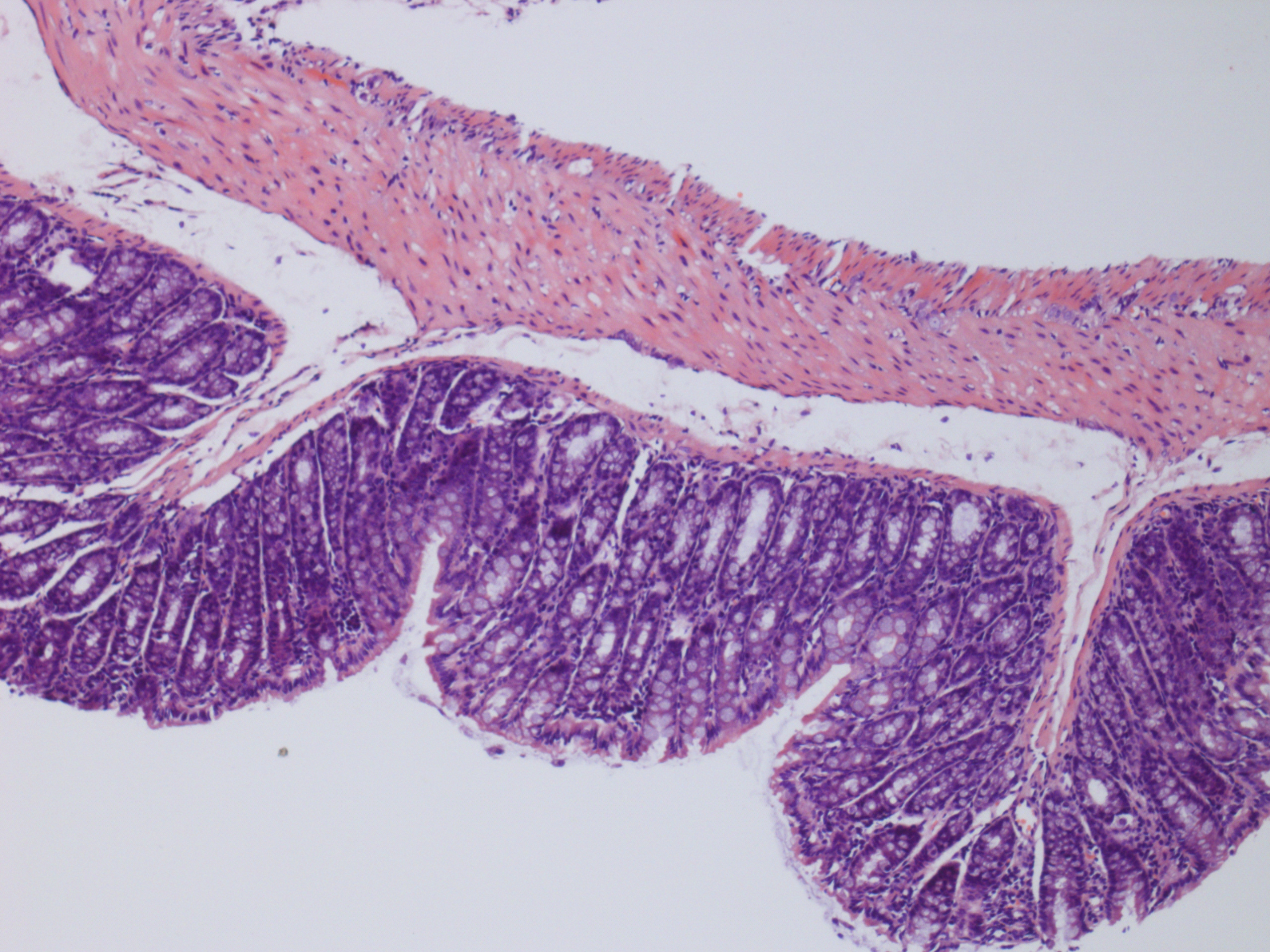

Supplement: Supplementary file 1 [file Image3.JPEG]

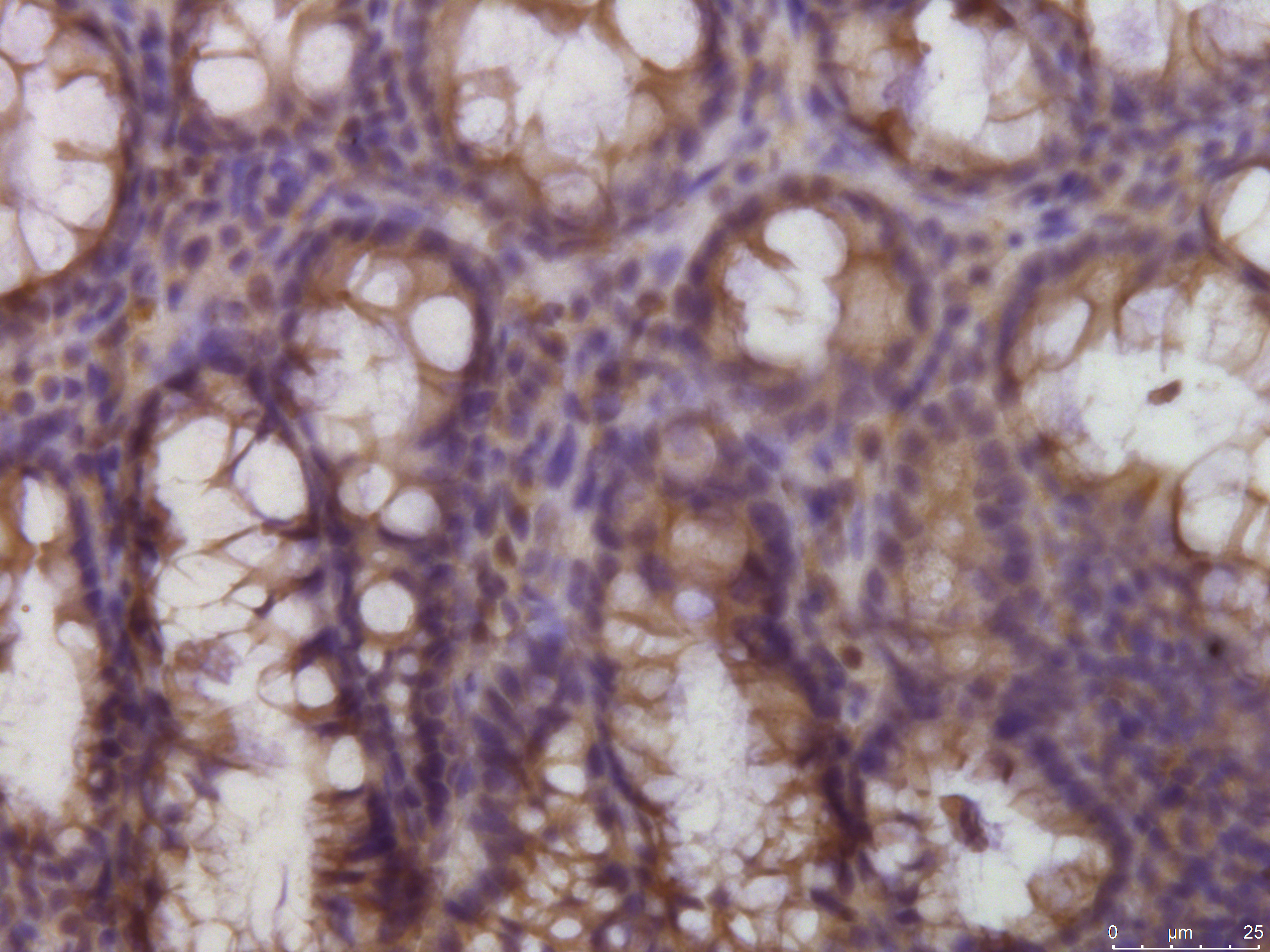

Supplement: Supplementary file 2 [file Image6.TIF]

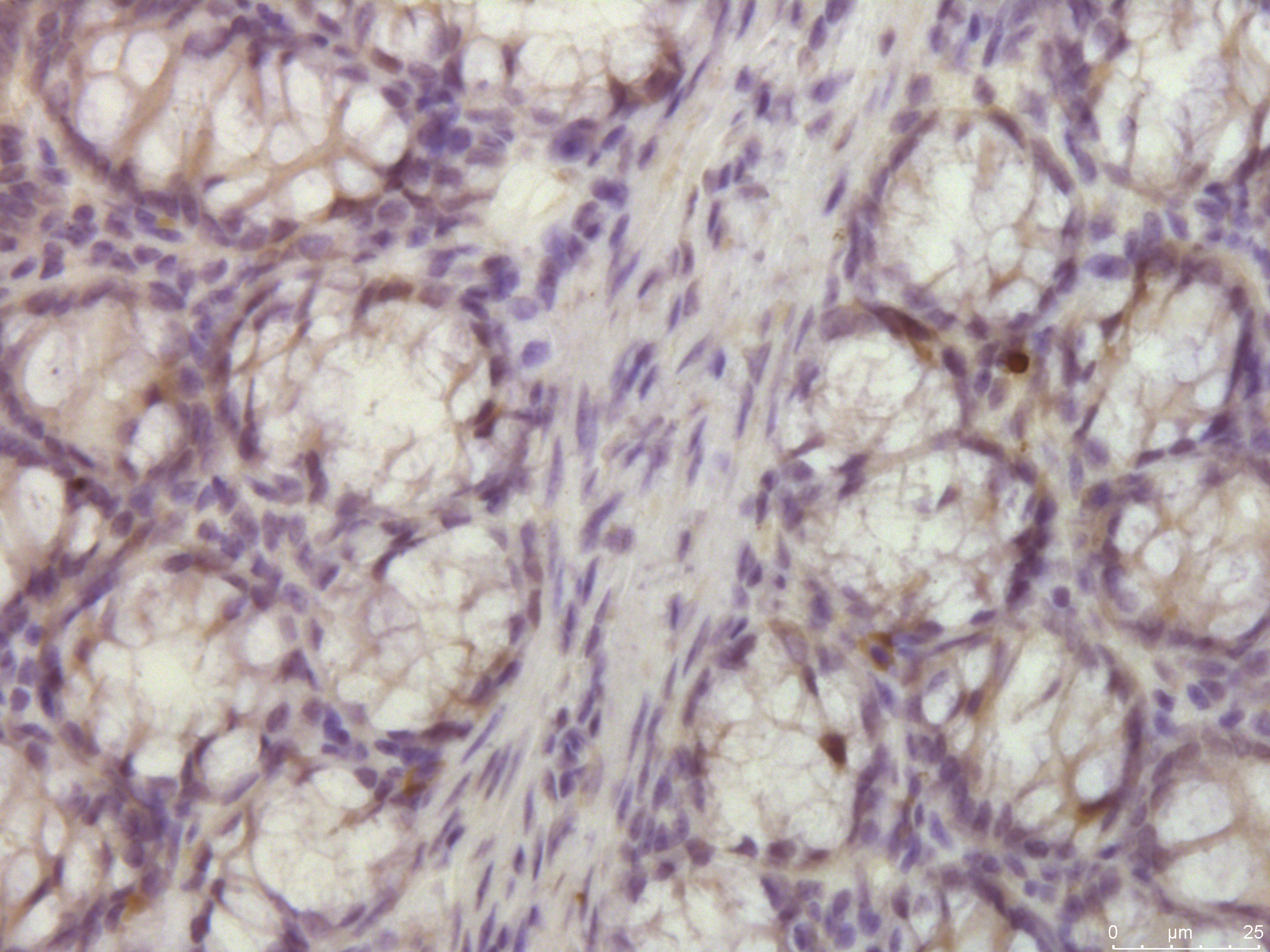

Supplement: Supplementary file 4 [file Image9.TIF]

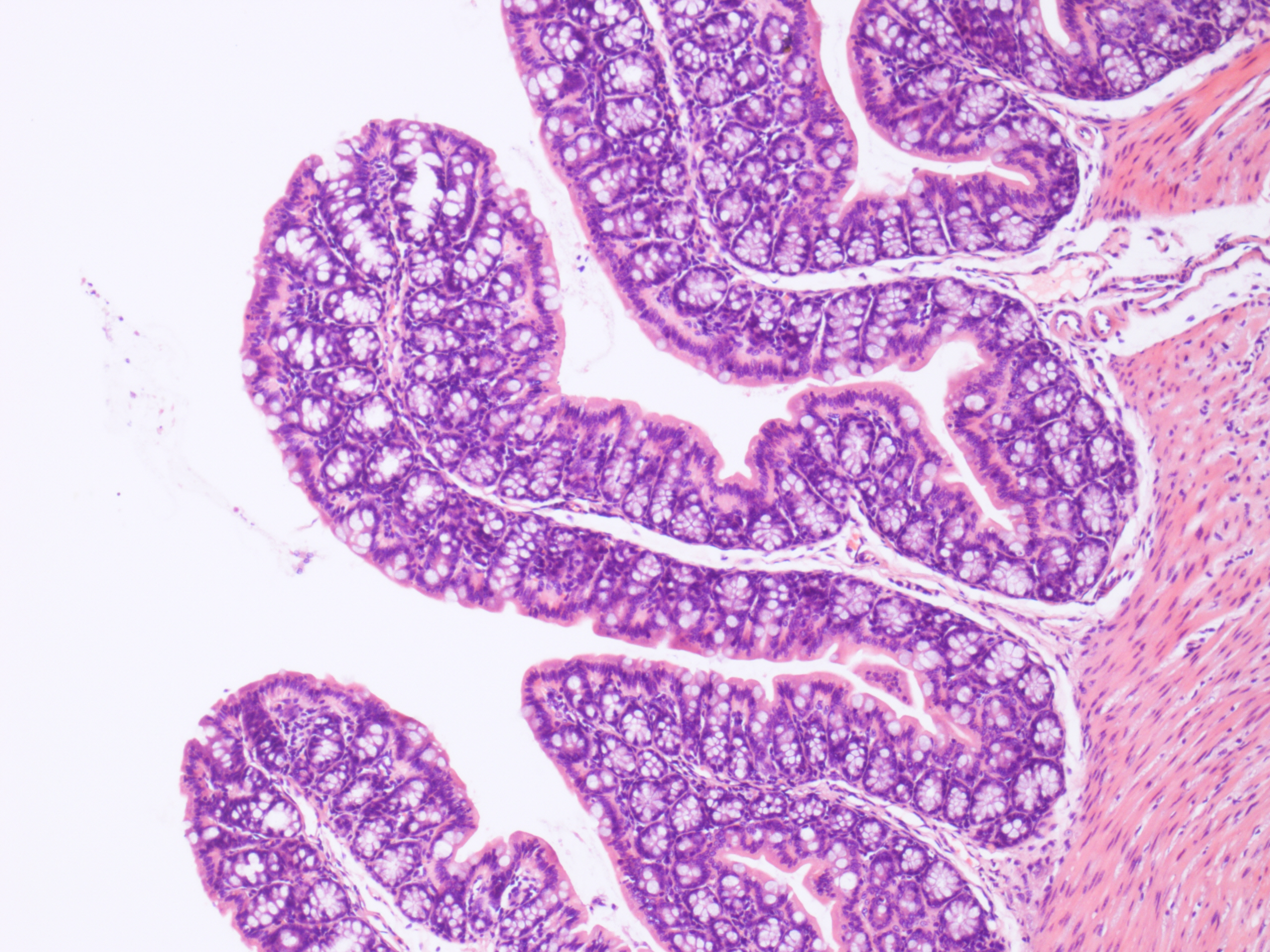

Supplement: Supplementary file 5 [file Image1.JPEG]

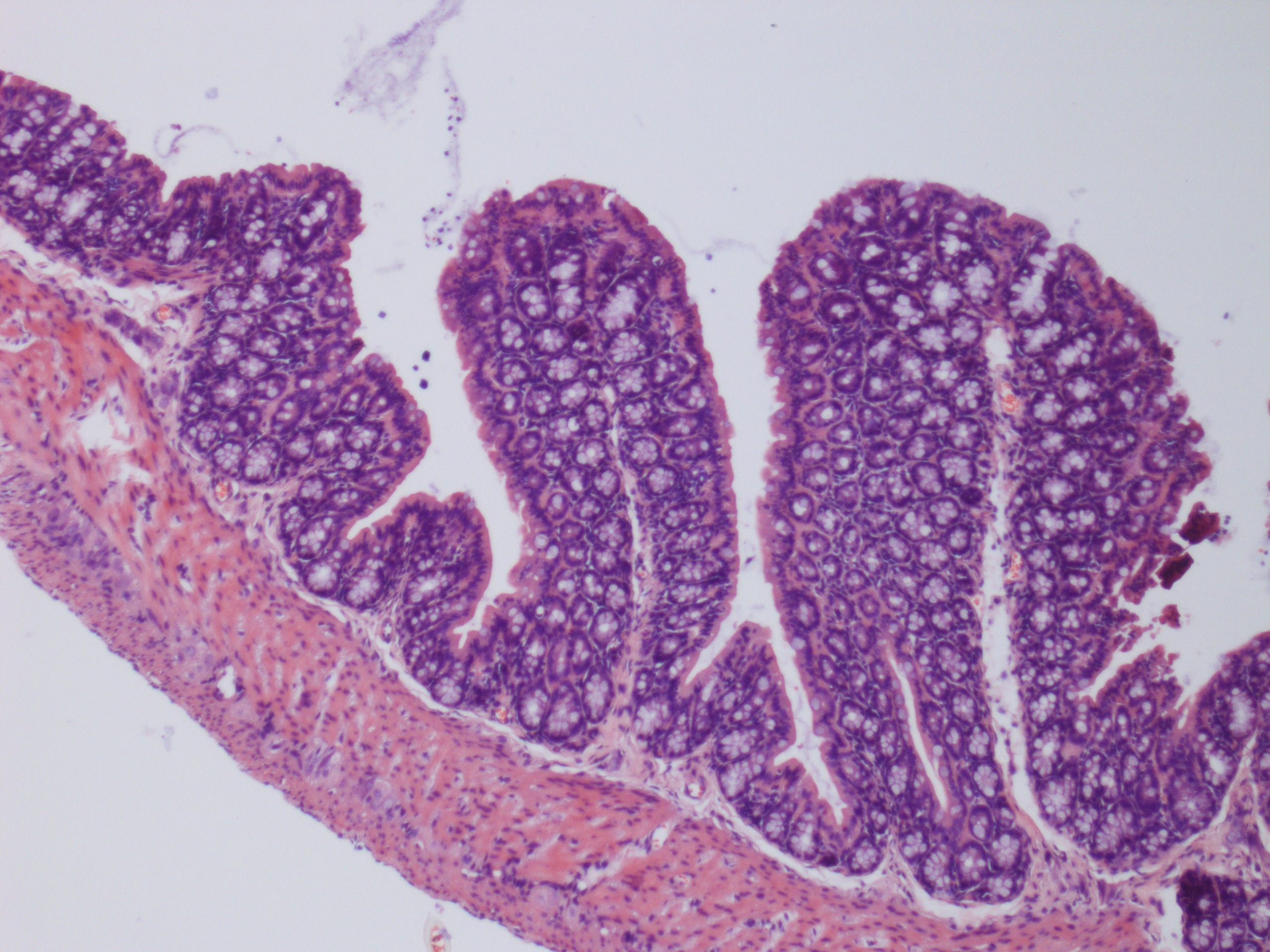

Supplement: Supplementary file 6 [file Image4.JPEG]

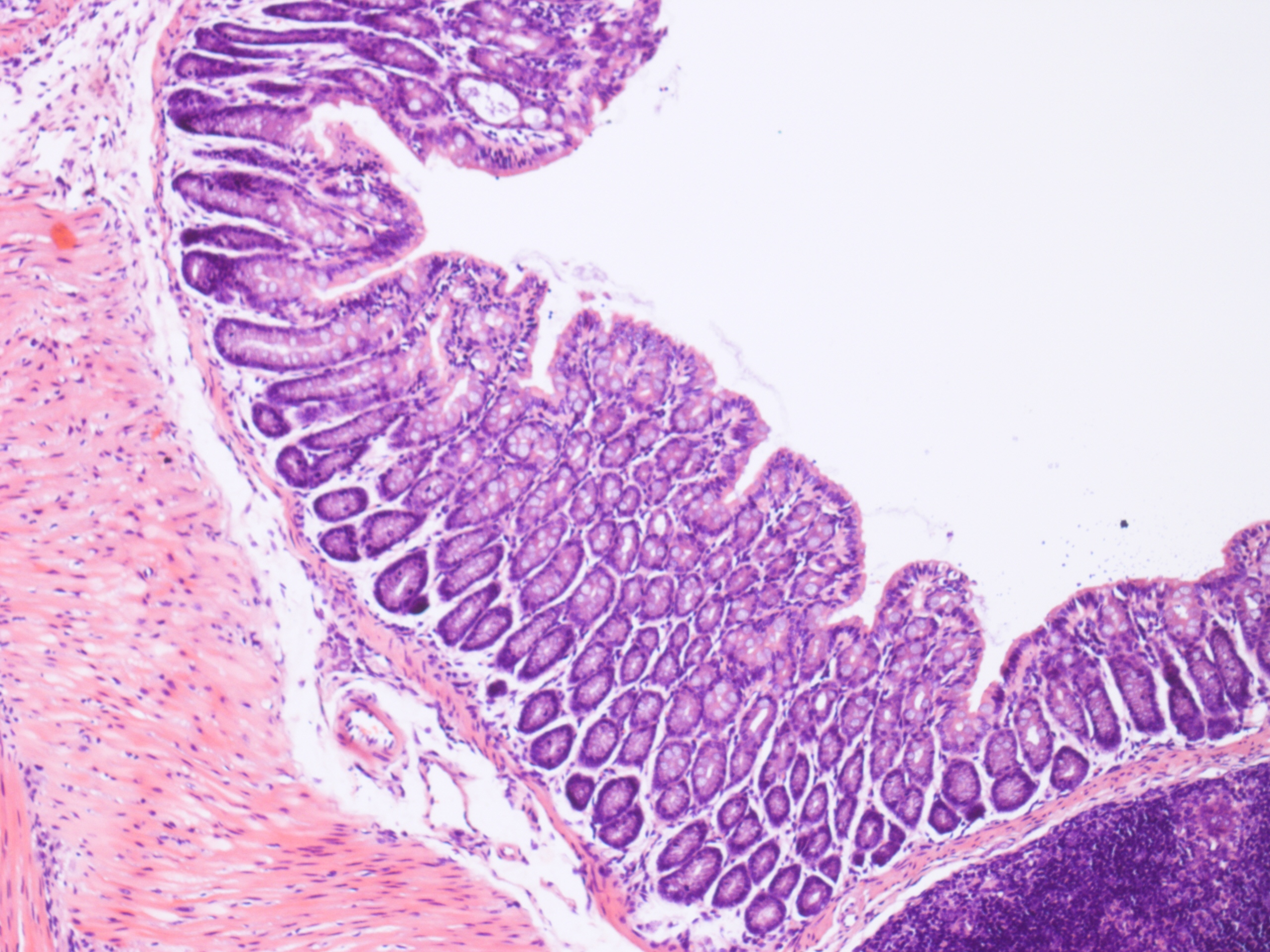

Supplement: Supplementary file 8 [file Image2.JPEG]

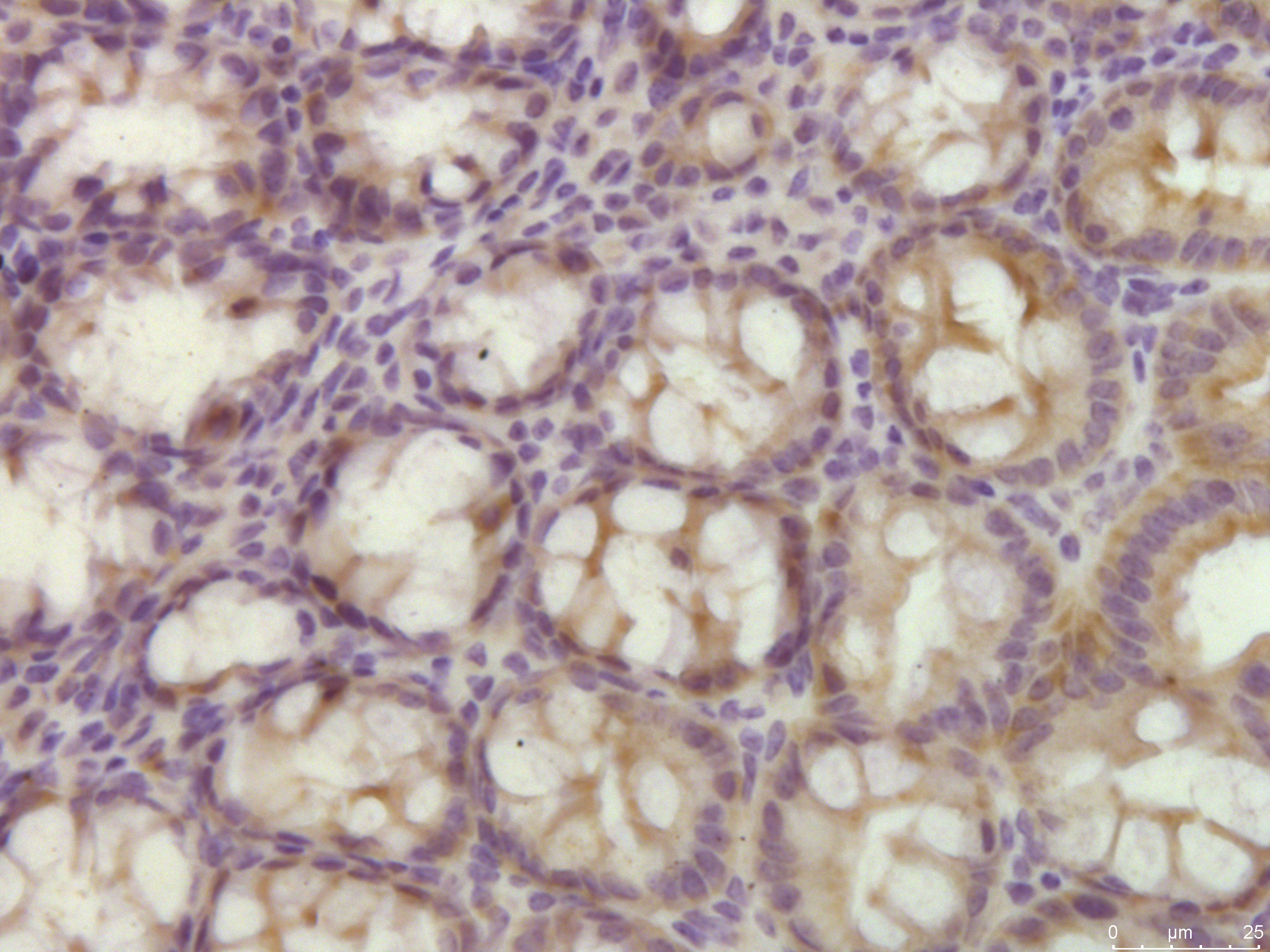

Supplement: Supplementary file 9 [file Image11.TIF]

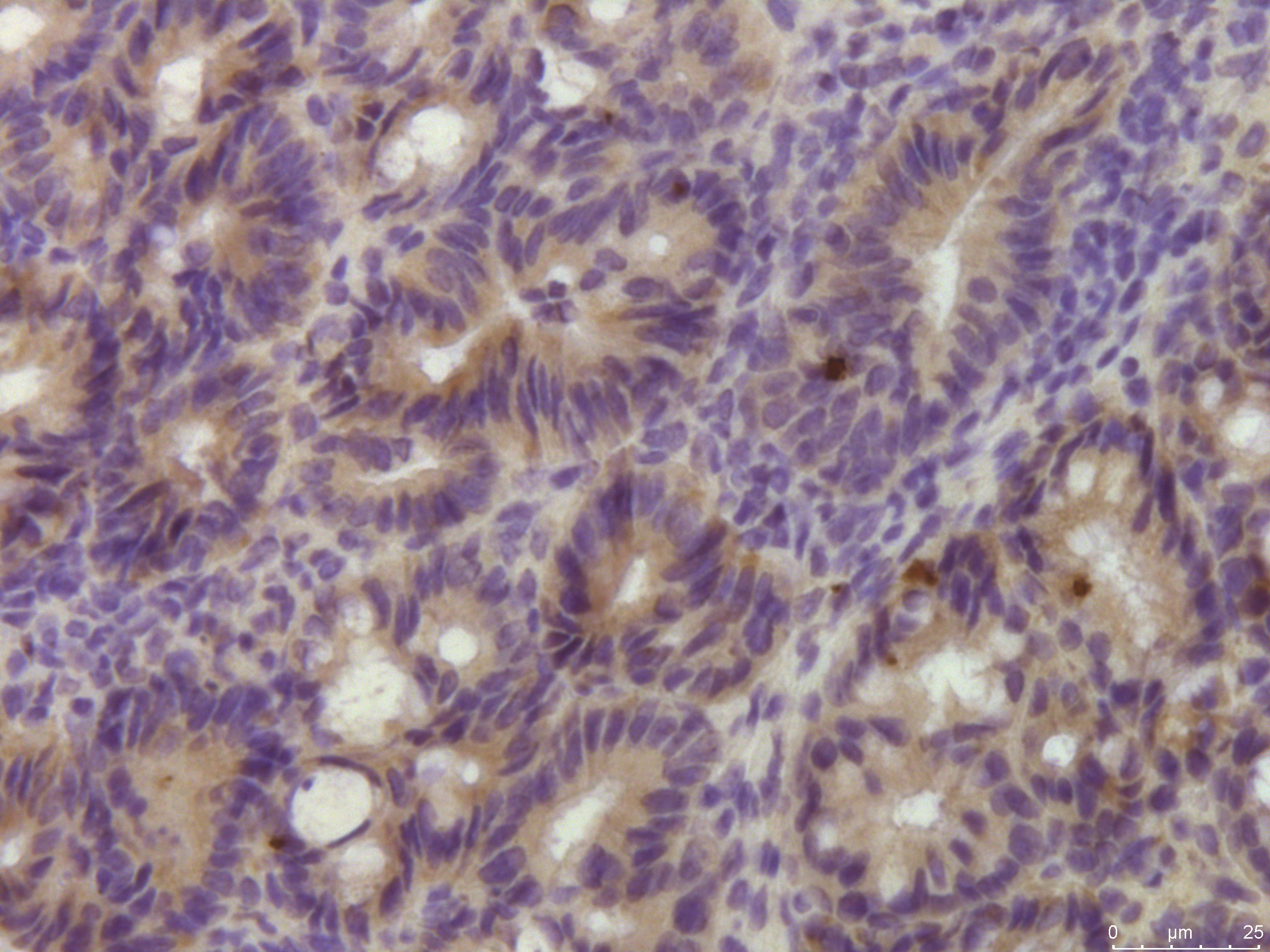

Supplement: Supplementary file 10 [file Image10.TIF]

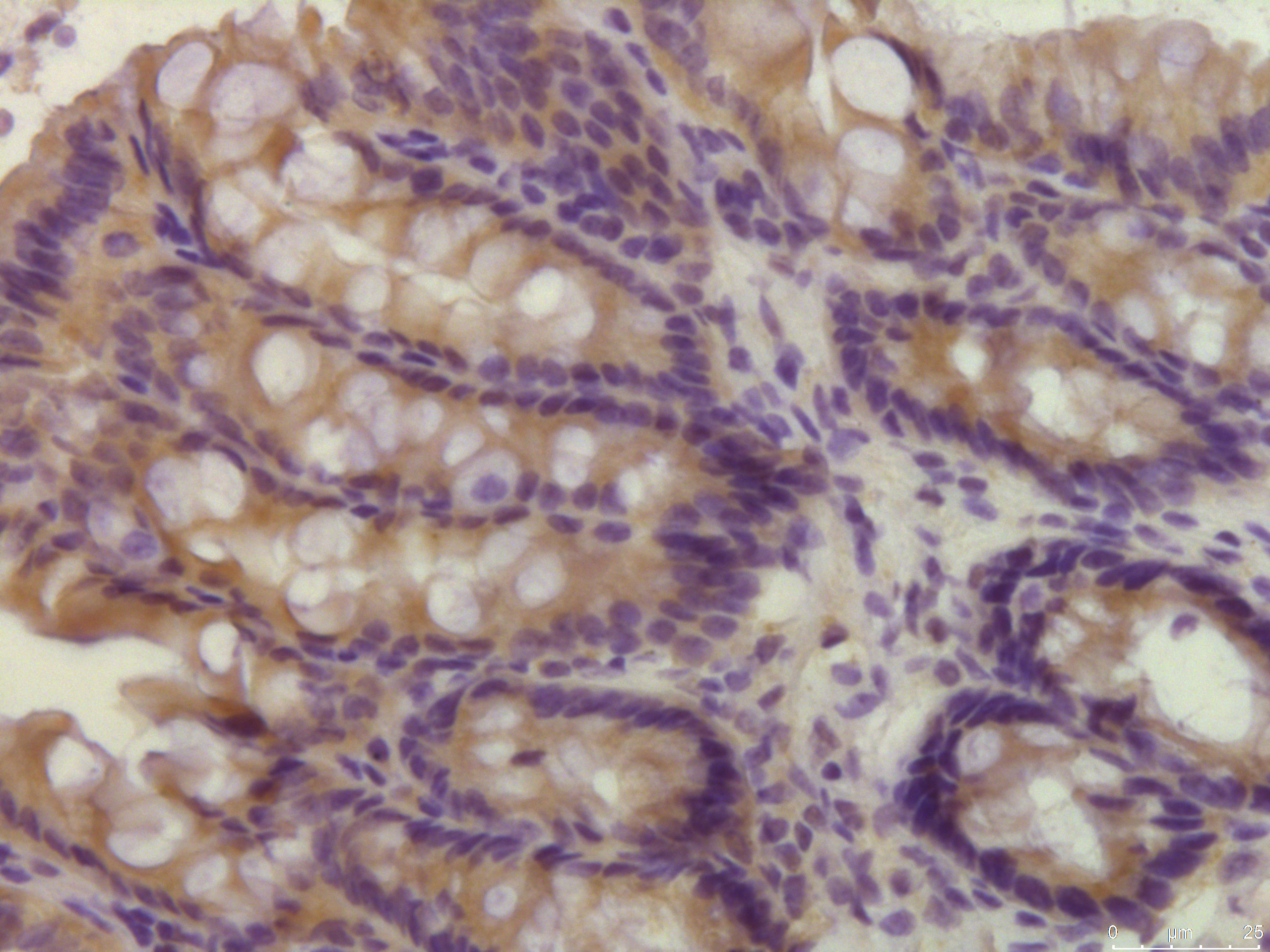

Supplement: Supplementary file 11 [file Image7.TIF]

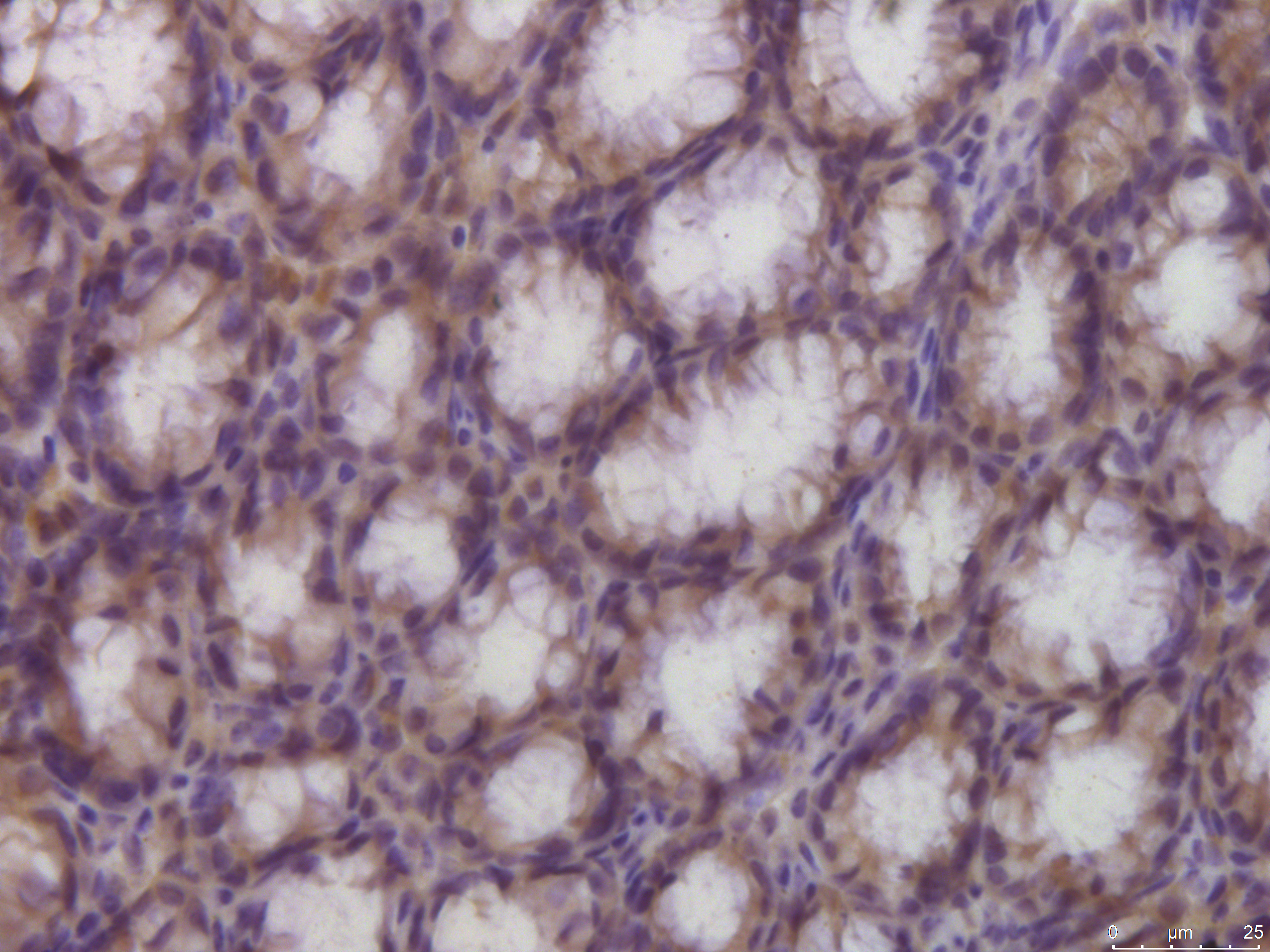

Supplement: Supplementary file 12 [file Image8.TIF]

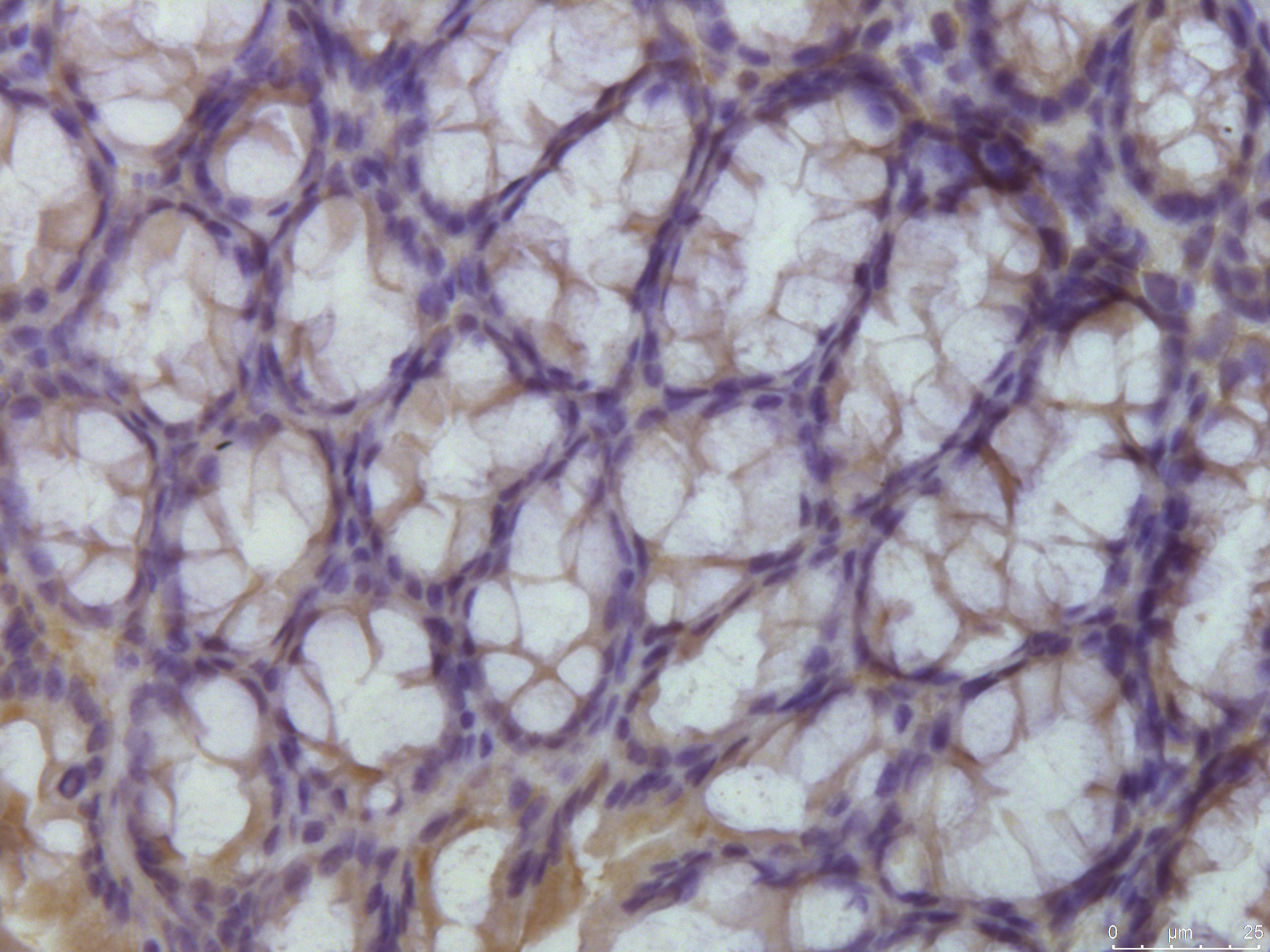

Supplement: Supplementary file 13 [file Image5.TIF]

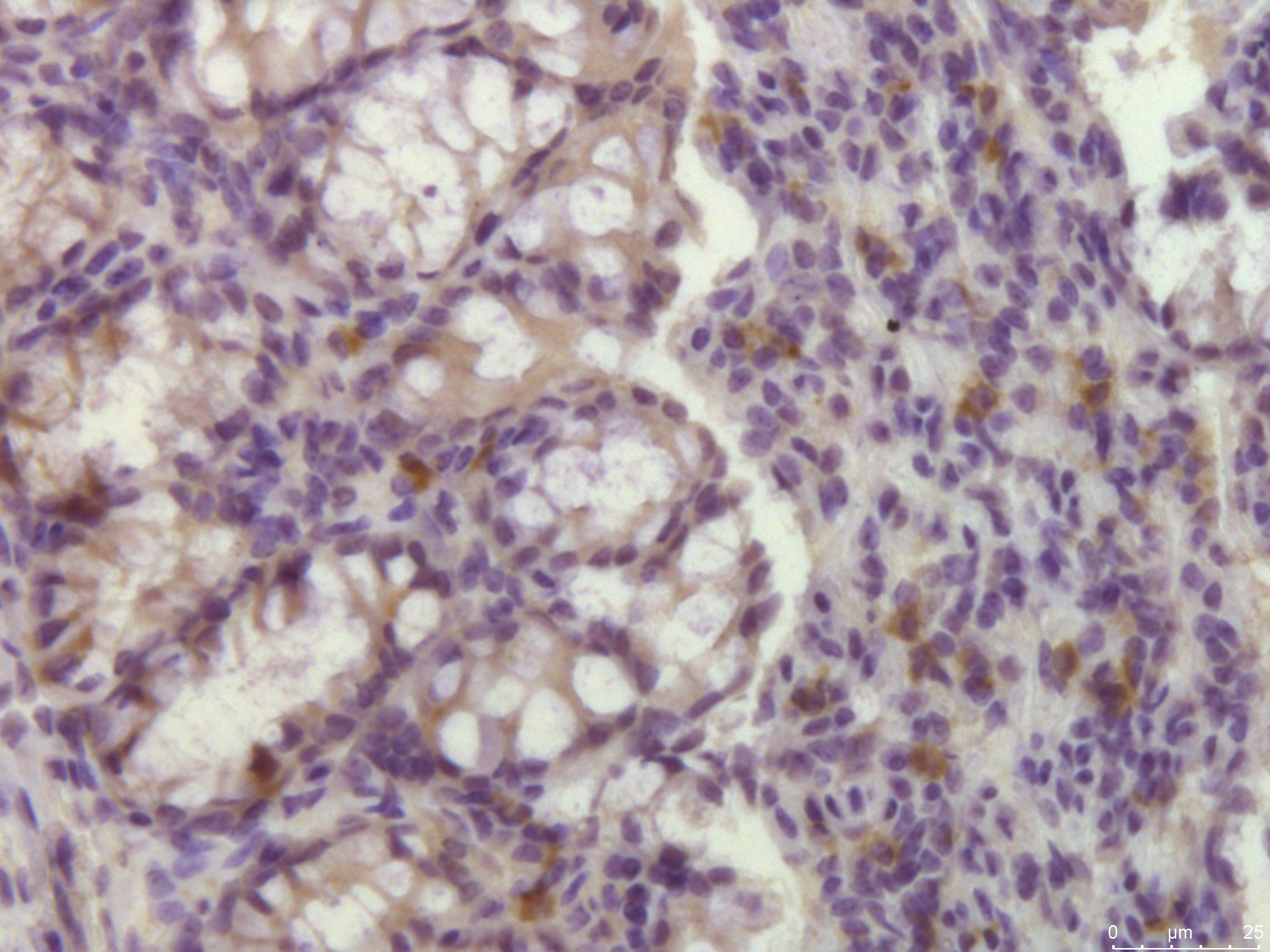

Supplement: Supplementary file 15 [file Image12.TIF]
